# Pre-weaning Ruminal Administration of Differentially-Enriched, Rumen-Derived Inocula Shaped Rumen Bacterial Communities and Co-occurrence Networks of Post-weaned Dairy Calves

**DOI:** 10.3389/fmicb.2021.625488

**Published:** 2021-02-26

**Authors:** Tansol Park, Laura M. Cersosimo, Wenli Li, Wendy Radloff, Geoffrey I. Zanton

**Affiliations:** ^1^USDA-Agricultural Research Service, Dairy Forage Research Center, Madison, WI, United States; ^2^Oak Ridge Institute for Science and Education, Oak Ridge, TN, United States

**Keywords:** adult rumen inoculation, rumen bacterial communities, protozoa, dairy calves, microbial network

## Abstract

Adult rumen fluid inoculations have been considered to facilitate the establishment of rumen microbiota of pre-weaned dairy calves. However, the sustained effects of the inoculations remain to be explored. In our previous study, 20 pre-weaned dairy calves had been dosed with four types of adult rumen inoculums [autoclaved rumen fluid, bacterial-enriched rumen fluid (BE), protozoal-enriched (PE), and BE + PE] weekly at 3 to 6 weeks of age. To verify the sustained effect of adult rumen inoculation, the rumen bacterial communities, fermentation characteristics, and animal performance measurements were measured after sacrifice from 20 post-weaned dairy bull calves (9 weeks of age). Ruminal pH tended to be lower in BE treated calves (*n* = 10). All PE treated calves had rumen ciliates (>10^4^ cells per ml of rumen fluid). PE treated calves had greater VFA concentrations (*P* = 0.052), lower molar proportions of isobutyrate (*P* = 0.073), and butyrate (*P* = 0.019) compared to those of control calves. No treatment differences were found in all animal performance measurements. Both PE and BE inocula increased bacterial species richness, Faith’s phylogenetic diversity, and Shannon’s index in rumen liquid fractions. However, the relative proportion of those bacterial taxa possibly transferred from the donor’s rumen was minor. Microbial network analysis showed different co-occurrence and mutually exclusive interactions between treatments of microbial inoculations. Collectively, adult rumen inoculations in pre-weaned dairy calves slightly altered the rumen bacteriome of post-weaned calves without changing fermentation and animal performance.

## Introduction

The rumen microbial community of an adult dairy cow consists of anaerobic bacteria, protozoa, fungi, archaea and harmless viruses. The bacteria, fungi, and protozoa convert nutrients into volatile fatty acids (VFA) as the main source of energy for the host, while the archaea utilize fermentation by-products to produce methane. Dairy calves are born with a sterile, undeveloped rumen, and the initial colostrum and subsequent milk they consume, bypasses the rumen via the esophageal groove to the abomasum (Church, 1976; Rey et al., 2014; Meale et al., 2016). At and after birth, dairy calves acquire fibrolytic bacteria and methanogenic archaea in their gastrointestinal tracts through contact with the vaginal, mammary gland, colostrum, and milk microbial communities (Vi et al., 2004; Guzman et al., 2015). With the onset of solid feed intake, increases in ruminal mass, papillae growth, and fermentation and further establishment of the rumen microbial ecology occur (Vi et al., 2004; Roth et al., 2009; Dill-Mcfarland et al., 2017).

Several studies have demonstrated the resiliency and host-specificity of the rumen microbial ecology of adult dairy cows, suggesting that the established rumen microbial environment is resistant to manipulative strategies (Jewell et al., 2015; Weimer, 2015; Malmuthuge and Guan, 2017). In contrast, the period of early ruminal development shows potential to be a favorable time to manipulate and direct the rumen environment (Yáñez-Ruiz et al., 2015). Direct-fed microbials, early nutrition, and artificial dosing with ruminal contents from adults have been utilized as strategies to direct the microbial ecology of the gastrointestinal tract of young ruminants and subsequent production performance (Schönhusen et al., 2003; Ishaq et al., 2015; Fouladgar et al., 2016; Zhang et al., 2017; Li et al., 2019). Li and co-authors found that separate ruminal wall microbial communities formed between dairy calves dosed with and without adult-derived rumen fluid (Li et al., 2019). These results were interpreted to support the manipulation of early, artificial dosing on the microbial community in ruminal wall. Additionally, Ishaq et al. demonstrated that moose-derived fibrolytic bacteria inoculation increased dietary efficiency of lambs (Ishaq et al., 2015).

In our previous study investigating the effect of adult-derived rumen fluid inoculation, two different microbial inocula (bacterial- or protozoal-enriched; BE or PE, respectively) were dosed in pre-weaned dairy calves to evaluate the effect of specific rumen microbiota on host growth responses, ruminal fermentation, and bacterial community composition (Cersosimo et al., 2019). We found that inoculation resulted in minor changes in some bacterial abundances and were accompanied with altered ruminal ammonia and butyrate concentrations, although calf health and growth were unaffected (Cersosimo et al., 2019). However, the effect of altered microbial community by inoculation might be accentuated after accelerated solid feeding that occurs after weaning. Furthermore, in our previous study, only 60% of the dairy calves treated with PE inoculum had a consistent rumen protozoa population pre-weaning (Cersosimo et al., 2019). Successful establishment of the protozoal population was hypothesized to induce shifts in the rumen microbiome and fermentation profile in post-weaned calves, though currently there is limited information about a potential carry-over effect. With the post-weaning period as the focus of this study, our objective was to investigate if the BE and PE microbial inoculations administered at 3–6 weeks of age during the pre-weaning period (Cersosimo et al., 2019) would affect rumen fermentation characteristics, rumen tissue (*e.g*., papillae length), and bacterial community structures of post-weaned, dairy bull calves (sacrificed at 9 weeks).

## Materials and Methods

### Experimental Design and Calf Management

This work is a continuation of our previously published work (Cersosimo et al., 2019). All animal procedures were approved by The University of Wisconsin’s Institutional Animal Care and Use Committee under protocol A005829. Holstein bull calves (*n* = 20) were enrolled into the study at birth over a 4-week period from July to August 2017. Calves were removed from their dam at birth, received colostrum within 4 h after birth, and were housed in individual calf hutches at the US Dairy Forage Research Farm in Prairie du Sac, WI. Calves were randomly assigned to a 2 × 2 factorial design with 4 different types of rumen inocula. Briefly, treatments previously described in Cersosimo et al., 2019, included 50 mL autoclaved, clarified rumen fluid (RF), PE, BE or 50 mL of each BE and PE. Five of the dairy calves were assigned to each treatment. Calves received 2.5 L pasteurized, antibiotic-free waste milk 3 × daily and were offered Vita Plus BSF 18 texturized calf starter (Vita Plus Corp., Madison, WI) at 6 d of age that contained shell corn, soybean meal, cottonseed hulls, kibbled corn, cane molasses, and heat processed soybeans. As-fed starter and refusals were measured on a daily basis from d 6 to 9 weeks of age. Calves were weaned at 7 weeks of age, thereafter their diet was only comprised of the texturized calf starter. Pre-weaned calves were orally dosed once per week with treatment inocula at 3 to 6 weeks of age. Dose administration was followed by 50 mL 0.7% sterile saline to clear the tube of residual inocula. Specific tubes were designated by inoculum type to avoid cross contamination of treatments.

### Sample Collection

The stomach compartments (reticulorumen, omasum, and abomasum) were harvested after euthanasia by penetrating captive bolt followed by exsanguination at 9 weeks of age at the University of Wisconsin Meat Science Laboratory. Each stomach compartment was isolated from each other with zip ties and weighed. Ruminal pH was measured *in situ* and whole rumen contents were collected from each rumen compartment and squeezed through four layers of cheesecloth to obtain a total of 50 mL of fluid for volatile fatty acid, ammonia, protozoal identification, and bacterial community analyses. After squeezing whole rumen contents through cheesecloth, 20–30 g of rumen solids were collected and stored at -80°C for bacterial community analyses. Complete ruminal contents were collected to measure rumen dry matter contents. Each rumen was washed to remove sand and any remaining feed particles before tissue sampling. A total of three 1 × 1 cm tissue samples were collected from the caudal ventral region of the rumen. The samples were stored in 10% formalin before measuring papillae length and number, and rumen wall width. Papillae length and rumen wall width were measured with digital calipers.

### Calf Measurements, Protozoal Counts, and Fermentation Characterization

Individual calf body length, weight, paunch and cardiac girths, wither and hip heights were measured once per week, from 1–9 weeks of age. Calf body measurements from 1–6 weeks of age were previously reported in Cersosimo et al., 2019.

A total of 5 mL of strained rumen fluid fixed in 50% formalin (v/v) at room temperature was collected for protozoal quantification, 5 mL for DNA extractions, and 5 mL with 0.1 mL 50% H_2_SO_4_ immediately frozen at -80°C for NH_3_ and VFA analyses, with the remaining 35 mL frozen and saved as extra rumen fluid at -80°C. A total of 4 mL of acidified rumen fluid was centrifuged at 30,000 × *g* at 4°C for 30 min and the supernatant was subsequently collected and stored at -20°C for VFA and NH_3_ analyses. Gas-liquid chromatography and Lachat methods, as previously described by Paula et al. (2018), were used to analyze VFA and NH_3_, respectively. Protozoa were detected using the microscopy methods outlined by Dehority (1984, 1993) and modified by Cersosimo et al. (2019).

### DNA Extraction and Sequencing

Rumen fluid, BE and PE inocula were thawed and centrifuged at 10,000 × *g* for 30 min at 4°C to retain the microbial pellet for the DNA extraction method (Yu and Morrison, 2004). For the rumen solids, a total of 30 mL sterile 0.9% NaCl (w/v) kept cold at 4°C was added to 10 g of rumen solids in a sterile Stomacher bag with a strainer to remove solid material (feed, hair, sand from bedding) and obtain microbial cells. The bag was placed and homogenized in a Stomacher (Seward, West Sussex, BN) on full speed for 2 min. and a total of 20 mL of the strained liquid was collected into a sterile conical tube and kept cold on ice. The liquid obtained from the solid sample was centrifuged at 500 × *g* for 15 min at 4°C to remove residual particles. The supernatant was centrifuged at 10,000 × *g* for 30 min at 4°C to obtain the microbial pellet for DNA extraction.

DNA was extracted from the microbial pellets obtained from each rumen fluid and solid fractions, and microbial inocula. DNA concentrations were quantified using the Broad Range kit for the Qubit ^®^Fluorometer (Invitrogen, San Diego, CA, United States). The methodologies and universal primers outlined previously (Kozich et al., 2013) were used to target the V4 hypervariable region of the bacterial 16S rRNA gene. PCR reaction details, thermal cycler conditions, and gel extraction information were previously described by Cersosimo et al., 2019. The pooled DNA library was sequenced, in-house, with the MiSeq 2 × 250 kit with 500 cycles (Illumina, San Diego, CA, United States) on an Illumina MiSeq. Note that the week 9 post-weaned samples were sequenced in the same sequencing run as samples collected from weeks 3–6 of age. The demultiplexed paired-end reads were processed using QIIME2 plugins (version 2019.10) (Bolyen et al., 2018). Quality filtering (Q-score ≥ 25), denoising, read merging, and chimeric sequence removal were sequentially done using the DADA2 plugin (Callahan et al., 2016). The resulting amplicon sequencing variants (ASVs) were taxonomically classified using the naïve Bayes taxonomy classifier pre-trained on Silva 16S databases (NR 132 version; clustered at 99% similarity). ASVs identified as unassigned, mitochondria, chloroplasts, or archaea were filtered out before downstream analysis. Major classified taxa, which were detected in over 50% of the samples at least one of the treatments, were discussed in this study.

### DNA Analyses

Alpha- and beta-diversity analyses were performed with the rarefied ASV table using the lowest sequence count (14,893 ASVs). Richness (observed ASVs and Chao1 estimates), Evenness, Faith’s phylogenetic diversity, Shannon’s index, and Simpson’s index were calculated from the rarefied ASV table. Beta-diversity shaped by different microbial inoculations was visualized using principal coordinates analysis (PCoA) plots based on the unweighted and weighted UniFrac distances using the QIIME2 emperor plugin (Vázquez-Baeza et al., 2013). Microbial metabolic functions were predicted from 16S ASVs using Phylogenetic Investigation of Communities by Reconstruction of Unobserved States 2 (PICRUSt2) (Douglas et al., 2019). Kyoto Encyclopedia of Genes and Genomes (KEGG) pathways were reconstructed from predicted KEGG ortholog profiles using the python script implemented in PICRUSt2. The effects on the overall functional profiles were examined based on the relative abundance of KEGG ortholog annotations and then principal components analysis (PCA) plots based on the Bray-Curtis similarity index was generated. The PCA plots were visualized using the R package ggfortify (Tang et al., 2016).

Microbial inoculation effects on the specific bacterial co-occurrence and mutual exclusion network were determined based on the compositional data of major bacterial genera, which were present in over 50% of the samples in at least one of the inoculated treatments. Correlations among major bacterial genera were determined using Sparse Co-occurrence Network Investigation for Compositional data (SCNIC) (https://github.com/shafferm/SCNIC) by computing Spearman correlation coefficients. Among the significant correlations (*P* < 0.05), specific microbial interactions at each inoculated treatment were defined according to their commonalities calculated using the R package, Co-expression Differential Network Analysis (CoDiNA) (Gysi et al., 2018). Additionally, the number of shared and exclusively found microbial taxa either in rumen liquid or solid between treated and control calves at phylum and genus levels were visualized using Venn diagrams.

### Statistical Analysis

Treatment groups were denoted as follows; with or without BE inoculation: BE(+) or BE(−), respectively, and with or without PE inoculation: PE(+) or PE(−), respectively. To compare alpha-diversity measurements between different ruminal fractions, PROC MIXED in SAS 9.3 (SAS Institute Inc., Cary, NC, United States) was used with the fixed effect of ages or ruminal fractions and random effect of calf. Animal performance measurements, fermentation characteristics, protozoal counts, and alpha diversity measurements were statistically analyzed using GLIMMIX procedure of SAS 9.3 with BE, PE and the interaction between BE and PE as fixed effects.

Relative abundance of both the classified microbial taxa and the predicted KEGG pathways were statistically analyzed by non-parametric Kruskal-Wallis test in R (v3.5.0). A PERMANOVA test implemented in PAST3 (Hammer et al., 2001) with 9,999 random permutations was used to check the significance of beta-diversity differences by ruminal fraction types (solid vs. liquid) and microbial inoculation types. Pearson correlation coefficients (correlation coefficient, | *r*| ≥ 0.5, *P* ≤ 0.05) between animal performance measurements, stomach compartment weights, fermentation characteristics, and the relative abundance of differentially abundant genera were determined using the PROC CORR procedure in SAS 9.3 and subsequently visualized using the corrplot package in R. Within each exclusive network selected by CoDiNA, the network statistics including the measurements of centrality (*i*.*e*., eigenvector centrality and authority) were calculated using the built-in plugins in Gephi (Bastian et al., 2009). Significance was declared at *P* ≤ 0.05 and trends at 0.05 < *P* ≤ 0.1.

## Results

### Stomach Compartment Weights, Fermentation Characteristics, and Protozoal Counts

Stomach compartment weights, ruminal dry matter%, ammonia, and total free amino acids concentrations did not differ by microbial inoculum type (Table 1). Likewise, body measurements and rumen papillae characteristics did not differ by microbial inoculum type (Supplementary Table 1). Omasum wet weights tended to be greater in PE(+) calves than PE(−) (*P* = 0.094). Ruminal pH tended to be lower in BE(+) calves than BE(−) (*P* = 0.079; Table 1). Proportions of ruminal butyrate (% of total VFA) were lower in PE(+) calves (5.57 ± 0.29%) than with PE(−) (7.95 ± 0.83%; *P* = 0.019). Calves treated with PE tended to have lower concentrations of isobutyrate than PE(−) (Table 1). Total ruminal VFA concentrations tended to be higher in PE(+) calves (73.71 ± 4.04 mM) than PE(−) (58.76 ± 5.76 mM; *P* = 0.052). No differences in total or individual VFA were observed between BE(+) and BE(−) calves. Only PE(+) treated calves had established rumen protozoal populations and mean protozoal counts were 4.34 ± 0.05 log10 counts per ml of rumen fluid. One calf treated with the PE inoculum had 1,000 holotrichs per ml of rumen fluid and entodiniomorphs, while the other 9 calves had entodiniomorphs exclusively.

**TABLE 1:** The main effects of microbial inoculations on the gut size, ruminal fermentation characteristics, and protozoal counts.

Measurements	BE		PE		SEM	*P*-values		
	+	-	+	-		BE	PE	BE × PE
**Wet weights (kg)**								
Rumen wet	7.99	7.70	7.93	7.77	0.445	0.750	0.861	0.150
Omasum	0.81	0.71	0.86	0.66	0.061	0.419	0.094	0.193
Abomasum	1.39	1.13	1.23	1.29	0.085	0.160	0.727	0.520
Rumen empty	1.93	1.71	1.81	1.83	0.088	0.248	0.930	0.365
**Ruminal environment**								
Total rumen DM (kg)	0.99	0.97	1.00	0.96	0.087	0.945	0.794	0.108
Rumen DM (%)	17.33	16.37	17.18	16.52	0.617	0.463	0.613	0.356
pH	6.38	6.54	6.38	6.53	0.047	0.079	0.110	0.839
Ammonia (mM)	6.55	5.40	5.13	6.83	0.942	0.563	0.396	0.401
Total free amino acids (mM)	2.30	2.16	2.09	2.37	0.098	0.487	0.178	0.383
Total VFA (mM)	63.90	68.57	73.71	58.76	3.829	0.523	0.052	0.314
Acetate (mol/100 mol)	51.63	52.19	53.96	49.85	1.248	0.826	0.121	0.809
Propionate	35.20	36.18	35.26	36.11	0.984	0.648	0.691	0.598
Isobutyrate	1.24	0.97	0.93	1.27	0.098	0.155	0.073	0.737
Butyrate	7.04	6.48	5.57	7.95	0.508	0.547	0.019	0.673
A:P ratio	1.51	1.47	1.57	1.41	0.073	0.770	0.296	0.693
Protozoa (log10 counts/ml)*	2.16	2.18	4.34	0	0.499	0.703	<0.001	0.703

*Protozoa log cell counts (counts for holotrichs in one rumen sample were excluded).

*DM, dry matter.*

*VFA, volatile fatty acids.*

*BE, bacterial-enriched rumen fluid.*

*PE, protozoal-enriched rumen fluid.*

*SEM, standard error or the mean.*

### Inoculation Effects on Alpha- and Beta-Diversity of Rumen Bacterial Community

Amplicon sequencing of 40 rumen samples from both the ruminal fractions resulted in a mean of 26,616 sequences per sample (Supplementary Table 2). High-quality ASVs averaging 25,053 per sample were obtained after quality- and taxa-filtering (ranging from 14,893 to 51,348 ASVs per sample) (Supplementary Table 2). Alpha- and beta-diversity analyses were done using a rarefied BIOM table at 14,893 ASVs per sample. Good’s coverage of all the samples was higher than 99.8%. In rumen fluid, species richness, phylogenetic diversity, and Shannon’s index were significantly higher in calves treated with both the BE(+) and PE(+) compared to their controls (*P* < 0.05) (Table 2). Furthermore, in rumen fluid, PE(+) calves tended to have more evenness (*P* < 0.1) and had significantly greater Simpson’s index than PE(−) (*P* < 0.05). In rumen solids, tendencies of greater species richness and phylogenetic diversity were observed between BE(+) and BE(−) calves (*P* < 0.1), while no alpha-diversity measurements were different between PE(+) and PE(−) calves. Alpha diversity measurements did not differ by fraction type except the evenness which was greater in the solid fraction (Supplementary Table 3). A greater number of ASVs were found in the PE inoculum compared to that of the BE inoculum (*P* < 0.05) accompanied with the tendency of greater Chao1 estimates in PE inoculum (*P* = 0.056; Supplementary Table 4).

**TABLE 2:** The main effects of microbial inoculations on alpha diversity measurements in different ruminal fractions.

Liquid								
Diversity measurements	BE		PE		SEM	*P*-values		
	+	-	+	-		BE	PE	BE × PE
Observed ASVs	183	144	186	141	8.7228	0.005	0.002	0.894
Chao1 estimates	193	148	193	148	9.4327	0.003	0.003	0.957
Evenness	0.654	0.614	0.658	0.611	0.0134	0.124	0.074	0.683
Faith’s phylogenetic diversity	13.99	11.684	14.114	11.559	0.5115	0.005	0.002	0.687
Shannon’s index	4.906	4.394	4.956	4.344	0.1387	0.038	0.016	0.847
Simpson’s index	0.918	0.884	0.924	0.877	0.0107	0.084	0.020	0.828

**Solid**								
**Diversity measurements**	**BE**		**PE**		**SEM**	***P*-values**		
	**+**	**-**	**+**	**-**		**BE**	**PE**	**BE × PE**

Observed ASVs	173	139	160	152	8.3916	0.054	0.605	0.898
Chao1 estimates	178	144	165	158	8.6867	0.064	0.676	0.846
Evenness	0.682	0.656	0.661	0.677	0.0120	0.295	0.513	0.359
Faith’s phylogenetic diversity	13.412	11.515	12.734	12.193	0.4745	0.053	0.559	0.839
Shannon’s index	5.059	4.658	4.825	4.892	0.1253	0.129	0.792	0.560
Simpson’s index	0.931	0.912	0.913	0.93	0.0082	0.263	0.327	0.739

*Good’s coverage was > 99.8% in all samples.ASV, amplicon sequencing variant.BE, bacterial-enriched rumen fluid.PE, protozoal-enriched rumen fluid.SEM, standard error or the mean.*

**FIGURE 1:**
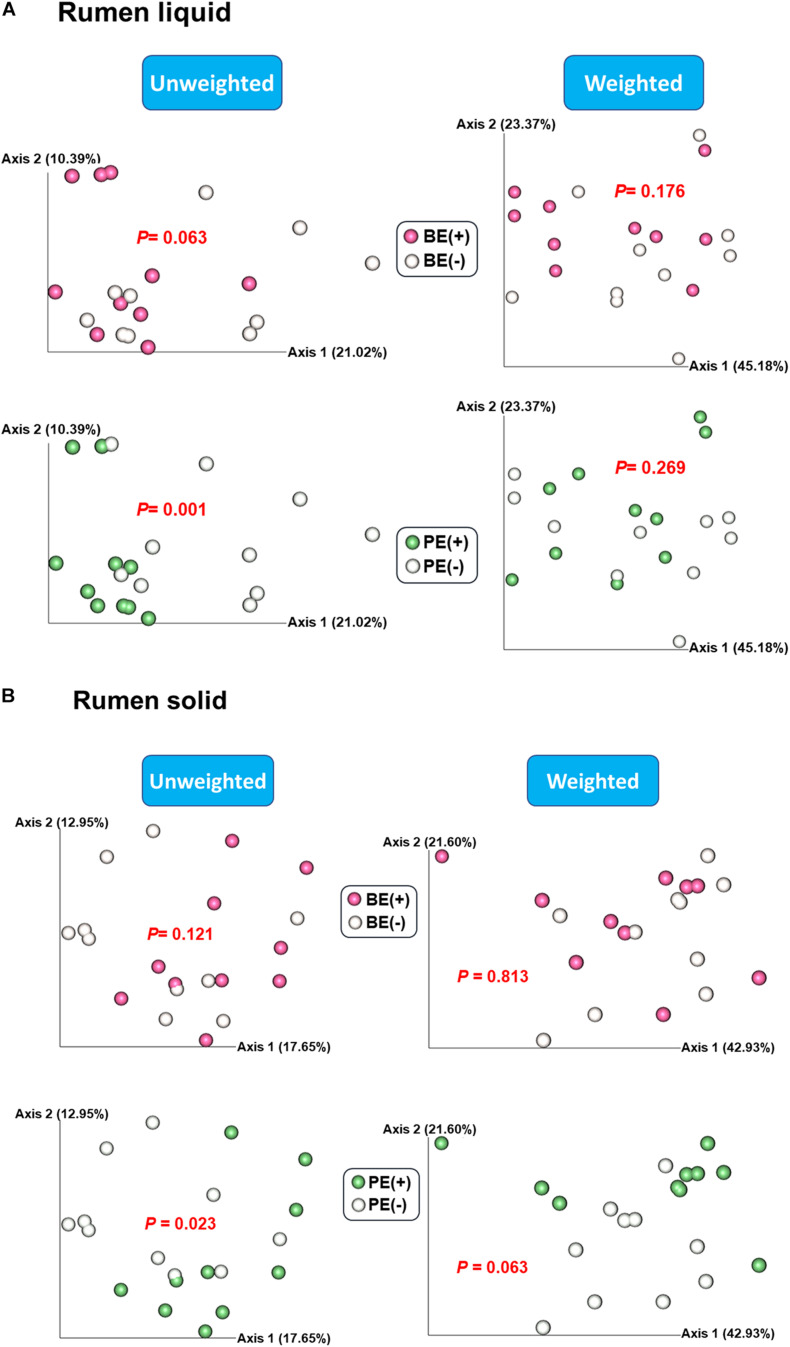
Principal coordinates analysis (PCoA) plot based on unweighted and weighted UniFrac distance matrices showing overall rumen bacterial microbiota distributions in **(A)** liquid and **(B)** solid fraction of dairy calves differed by microbial inoculations with two types of inoculums (BE and PE).

Based on the unweighted (qualitative) and weighted (quantitative) Unifrac distance matrices, including the impact of phylogeny, BE and PE affected overall bacterial community qualitatively in rumen fluids, but not quantitatively (*P* < 0.1; Figure 1A). In rumen solids, PE altered the overall bacterial community (*P* = 0.023 for unweighted UniFrac; *P* = 0.063 for weighted UniFrac) while BE had no significant impact (Figure 1B). Two different ruminal fractions had significantly different overall bacterial community, qualitatively and quantitatively (*P* < 0.01; Supplementary Figure 1).

### Inoculation Effects on the Existence and Composition of Rumen Bacterial Community

**FIGURE 2:**
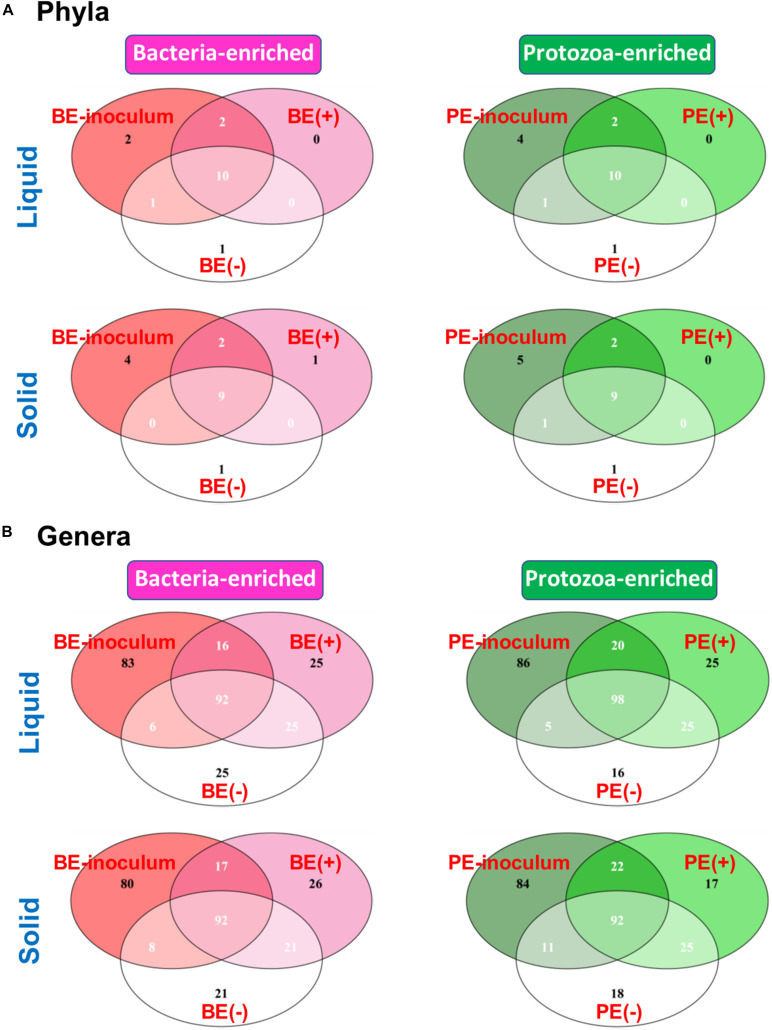
Venn Diagram depicting the number of shared and exclusive bacterial **(A)** phyla or **(B)** genera by microbial inoculations at either rumen liquid or solid fractions.

The Venn diagram showed shared bacterial taxa between donor inoculum and two treatment groups by either BE or PE inoculation (Figure 2). In rumen liquid, 10 phyla were shared among the respective inoculum sample, treated- and non-treated calves in both the BE and PE inoculations. In rumen solid, nine phyla were shared among these three groups (*i.e*., inoculum, treated- and non-treated) in both the BE and PE inoculations. Two phyla were shared only between inoculum and inoculum-treated calves regardless of the ruminal fractions and inoculum types, and Chloroflexi, Lentisphaerae, Kiritimatiellaeota, and Patescibacteria were the phyla belonging to that category. The phylum Verrucomicrobia was exclusively found in one, non-treated calf. At the genus level, over 90 genera were shared by three groups in both the BE and PE inoculations. Invariably, calves receiving either the PE or BE shared more phyla and genera than control groups. More than 80 genera were exclusively found in inocula, but those genera comprised only 4.44 to 5.88% of overall abundance in both the inocula. Furthermore, exclusively found genera by microbial inoculation treatments occupied a minor portion regardless of the ruminal fractions and inoculum types (< 1% of average relative abundance). Erysipelotrichaceae UCG-007 and Family XIII AD3011 group were the only major genera (present in at least 50% of the samples) exclusively found in the solid fraction of BE treatments and in the liquid fraction of PE treatments, respectively. The number of detected taxa between rumen liquid and solid fractions was also shown in Supplementary Figure 2.

An opposite trend of the relative abundance of the phyla Fibrobacteres and Proteobacteria in rumen fluid was observed by BE. The bacterial phylum Fibrobacteres was dominant in BE(+) calves [BE(+): 0.21 ± 0.08% and BE(−): 0.06 ± 0.03%, *P* = 0.02], whereas the relative abundance of Proteobacteria was higher in BE(−) calves [BE(+): 13.90 ± 3.63% and BE(−): 28.29 ± 4.94%, *P* = 0.03; Table 3A]. The same trend for both phyla was observed in rumen solids by PE [for Fibrobacteres, PE(+): 3.42 ± 0.94% and PE(−): 0.61 ± 0.24%, *P* = 0.02; for Proteobacteria, PE(+): 3.20 ± 1.31% and PE(−): 10.03 ± 2.40%, *P* = 0.03; Table 3B].

**TABLE 3:** Differentially abundant taxa by microbial inoculations in liquid and solid fraction.

A. Differentially abundant phyla (Liquid)						
Enrichment	Taxon	Relative abundance (%)^∗^		SEM	*P*-value	
		BE(+)	BE(−)			
BE(+) enriched	*Fibrobacteres*	0.209	0.057	0.0440	0.022	
BE(−) enriched	*Proteobacteria*	13.899	28.286	3.4100	0.034	

**B. Differentially abundant phyla (Solid)**						

**Enrichment**	**Phyla**	**Relative abundance (%)**		**SEM**	***P*-value**	
		**PE(+)**	**PE(−)**			

PE(+) enriched	*Fibrobacteres*	3.416	0.609	0.5716	0.016	
PE(−) enriched	*Proteobacteria*	3.204	10.026	1.5421	0.034	

**C. Differentially abundant genera (Liquid)**						
**Enrichment**	**Phyla**	**Genera**	**Relative abundance (%)**		**SEM**	***P*-value**
			**BE(+)**	**BE(−)**		

BE(+) enriched	*Bacteroidetes*	F082 URB	0.158	0.090	0.0306	0.023
		*Rikenellaceae* U29-B03	0.136	0.070	0.0225	0.040
	*Fibrobacteres*	*Fibrobacter*	0.209	0.057	0.0440	0.022
	*Firmicutes*	*Lachnoclostridium* 1	0.013	0.002	0.0027	0.041
		*Lachnospira*	0.088	0.050	0.0112	0.049
		*Lachnospiraceae* UCG-010	0.006	0.001	0.0012	0.027
		*Erysipelotrichaceae* UCG-007	0.209	0.010	0.0622	0.048

BE(−) enriched	*Proteobacteria*	*Succinivibrionaceae* UN	5.135	16.630	3.0559	0.041

**Enrichment**	**Phyla**	**Genera**	**PE(+)**	**PE(−)**	**SEM**	***P*-value**

PE(+) enriched	*Actinobacteria*	*Coriobacteriales* UN	0.029	0.005	0.0049	0.010
	
	*Bacteroidetes*	*Prevotellaceae* UCG-001	0.680	0.462	0.1366	0.049
	
		*Bacteroidales* URB	0.052	0.027	0.0101	0.025
		*Bacteroidales* UN	0.020	0	0.0057	0.036
	
	*Firmicutes*	*Christensenellaceae* R-7 group	0.131	0.044	0.0201	0.008
		*Clostridiales* vadinBB60 group UN	0.041	0.023	0.0113	0.037
		*Defluviitaleaceae* UCG-011	0.015	0.003	0.0033	0.014
		Family XIII AD3011 group	0.024	0	0.0043	0.005
		*Acetitomaculum*	1.787	0.152	0.4148	0.013
		*Butyrivibrio* 2	0.028	0.002	0.0042	0.002
		*Coprococcus* 1	0.214	0.010	0.0611	0.050
		*Lachnospiraceae* UCG-008	0.046	0.013	0.0073	0.014
		*Syntrophococcus*	0.671	0.346	0.0742	0.008
		*Clostridiales* UN	0.055	0.010	0.0095	0.009
	
	*Synergistetes*	*Synergistes*	0.029	0.003	0.0054	0.009

**D. Differentially abundant genera (Solid)**						

**Enrichment**	**Phyla**	**Genera**	**Relative abundance (%)**			
			**BE(+)**	**BE(−)**	**SEM**	***P*-value**

BE(+) enriched	*Bacteroidetes*	F082 URB	0.739	0.299	0.1136	0.034
		*Rikenellaceae* U29-B03	0.102	0.029	0.0201	0.042
	
	*Firmicutes*	*Erysipelotrichaceae* UCG-007	0.133	0	0.0456	0.005

PE(+) enriched	*Bacteroidetes*	*Bacteroidales* bacterium Bact_22	0.220	0.061	0.0698	0.032
	
	*Fibrobacteres*	*Fibrobacter*	3.416	0.609	0.5716	0.016
	
	*Firmicutes*	*Lachnospiraceae* UCG-008	0.025	0.003	0.0049	0.005
		*Eubacterium xylanophilum* group	0.114	0.036	0.0300	0.048
	
PE(−) enriched	*Actinobacteria*	*Eggerthellaceae* UN	0	0.008	0.0017	0.013
	
	*Bacteroidetes*	*Prevotella* 9	0.877	2.063	0.5122	0.023
	
	*Firmicutes*	*Roseburia*	0.015	0.066	0.0129	0.035
		*Ruminococcaceae* UCG-004	0.014	0.038	0.0053	0.029
	
	*Proteobacteria*	*Succinivibrionaceae* UN	1.987	8.974	1.5126	0.049

*Calculated based on the normalized read count BIOM tables.

*UN, unclassified; URB, uncultured rumen bacterium; UCG, uncultured genus-level group.*

*BE, bacterial-enriched rumen fluid.*

*PE, protozoal-enriched rumen fluid.*

*SEM, standard error or the mean.*

By PE, the abundance of *Fibrobacter* and Lachnospiraceae UCG-008 in the solid fraction was also dominant in PE(+) calves compared to that of PE(−) calves as shown in the liquid fraction. In the solid fraction, the abundance of Bacteroidales bacterium Bact_22 and *Eubacterium xylanophilum* group was greater in PE(+) calves compared to that of PE(−) calves, while the opposite distribution was found in the abundance of five genera (unclassified genus of Eggerthellaceae, *Prevotella* 9, *Roseburia*, Ruminococcaceae UCG-004, and unclassified genus of Succinivibrionaceae) by PE.

To show the preferential environmental niches of major bacterial phyla and genera, a comparison of their relative abundance between liquid and solid fractions was also analyzed (Supplementary Table 5). The bacterial phylum Proteobacteria and 14 genera were dominant in the liquid fraction (*P* < 0.05). A greater abundance of the three phyla (Bacteroidetes, Fibrobacteres, and Spirochaetes) and 14 genera were detected in the solid fraction compared to that of the liquid fraction (*P* < 0.05; Supplementary Table 5).

In the liquid fraction, two uncultured genera within the phylum Bacteroidetes (F082 uncultured rumen bacterium and Rikenellaceae U29-B03), *Fibrobacter*, and four genera within Firmicutes (Lachnoclostridium 1, *Lachnospira*, Lachnospiraceae UCG-010, and Erysipelotrichaceae UCG-007) were differentially abundant in BE(+) calves, whereas unclassified genus of Succinivibrionaceae was the only genus abundant in BE(−) calves compared to their counterparts. Comparing by PE, 15 genera belonging to four different phyla were differentially abundant in PE(+) calves, but there were no taxa significantly abundant in PE(−) calves were found (Table 3C). In the solid fraction, the relative abundance of F082 uncultured rumen bacterium, Rikenellaceae U29-B03, and Erysipelotrichaceae UCG-007 showed same distribution between BE(+) calves and BE(−) calves as determined in the liquid fraction (Table 3D).

### Differential Network Analysis

**FIGURE 3:**
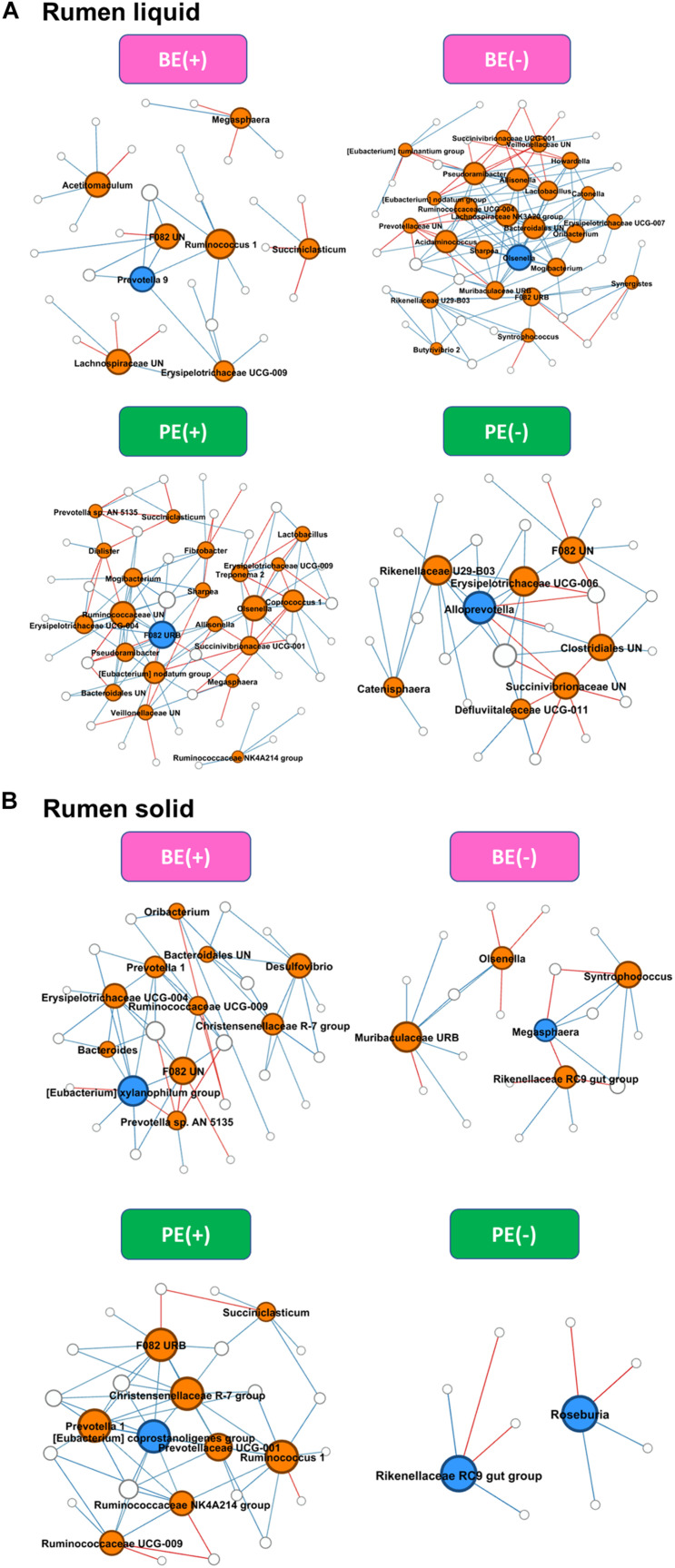
Exclusive co-occurrence and mutual exclusion microbial network either at **(A)** rumen liquid and **(B)** rumen solid. Node color represents exclusive microbial nodes (orange) and keystone taxa (blue) selected based on the betweenness centrality, authority, and eigenvector centrality measurements within each exclusive network. Edge color represents co-occurrence (blue) or mutual exclusive (red) interactions. Edge thickness was adjusted based on the absolute value of the correlation coefficients of each interaction.

Differential network analysis identified 36 and 52 nodes with 35 and 136 significant interactions among the major genera in rumen liquid between BE(+) and BE(−) calves, respectively (Figure 3). A total of 56 and 31 microbial nodes with 116 and 47 significant interactions were exclusively identified in rumen liquid bacteria of PE(+) and PE(−) calves, respectively. While a total of 31 and 22 nodes with 51 and 25 significant interactions from rumen solids consisted of the exclusive networks between BE(+) and BE(−) calves. A total of 28 and 10 nodes with 59 and 8 significant interactions from rumen solid fraction of PE inoculated calves were exclusively found either in microbial networks of PE(+) and PE(−) calves, respectively. Based on the centrality measurements, four bacterial genera in the rumen liquid [*Prevotella* 9 for BE(+), *Olsenella* for BE(−), F082 uncultured rumen bacterium for PE(+), *Alloprevotella* for PE(−)] and five bacterial genera in the rumen solid [*Eubacterium xylanophilum* group for BE(+), *Megasphaera* for BE(−), *Eubacterium coprostanoligenes* for PE(+), Rikenellaceae RC9 gut group and *Roseburia* for PE(−)] were denoted as keystone genera in the exclusive microbial networks of each microbial inoculation treatment (Table 4). Among those keystone microbial nodes at each specific network, the genera *Megasphaera* and *Eubacterium coprostanoligenes* group were liquid-dominant, while three bacterial genera (F082 uncultured rumen bacterium, *Eubacterium xylanophilum* group, and Rikenellaceae RC9 gut group) were solid-dominant (Supplementary Table 5).

**TABLE 4:** Exclusive network statistics by microbial inoculations either in rumen liquid or solid fraction.

**Liquid**				
	**BE(+)**	**BE(−)**	**PE(+)**	**PE(−)**

Nodes	36	52	56	31

Total edges	35	136	116	47

Positive	25	109	78	34

Negative	10	27	38	13

Positive (%)	71.43	80.15	67.24	72.34

Negative (%)	28.57	19.85	32.76	27.66

Abundance of exclusive node (%)	22.22	48.08	39.29	25.81

Network Diameter	4	7	6	6

Graph Density	0.056	0.103	0.075	0.101

Modularity	0.730	0.496	0.526	0.442

No. of communities	7	4	6	4

Average Clustering Coefficient	0.203	0.559	0.466	0.450

Best ‘Centrality’ node*	*Prevotella* 9	*Olsenella*	F082 URB	*Alloprevotella*

**Solid**				

	**BE(+)**	**BE(−)**	**PE(+)**	**PE(−)**

Nodes	31	22	28	10

Total edges	51	25	59	8

Positive	43	16	53	4

Negative	8	9	6	4

Positive (%)	84.31	64	89.83	50

Negative (%)	15.69	36	10.17	50

Abundance of exclusive node (%)	35.48	22.73	32.14	20

Network Diameter	9	4	6	2

Graph Density	0.110	0.108	0.156	0.178

Modularity	0.496	0.557	0.363	0.500

No. of communities	4	4	4	2

Average Clustering Coefficient	0.464	0.502	0.533	0

Best ‘Centrality’ node*	*Eubacterium*	*Megasphaera*	*Eubacterium*	*Rikenellaceae* RC9 gut
	*xylanophilum* group		*coprostanoligenes*	group & *Roseburia*
			group	

*Selected based on both the eigenvector centrality and authority.

*BE, bacterial-enriched rumen fluid.*

*PE, protozoal-enriched rumen fluid.*

*URB, uncultured rumen bacterium.*

### The Effect of Inoculation on the Microbial Functions

**FIGURE 4:**
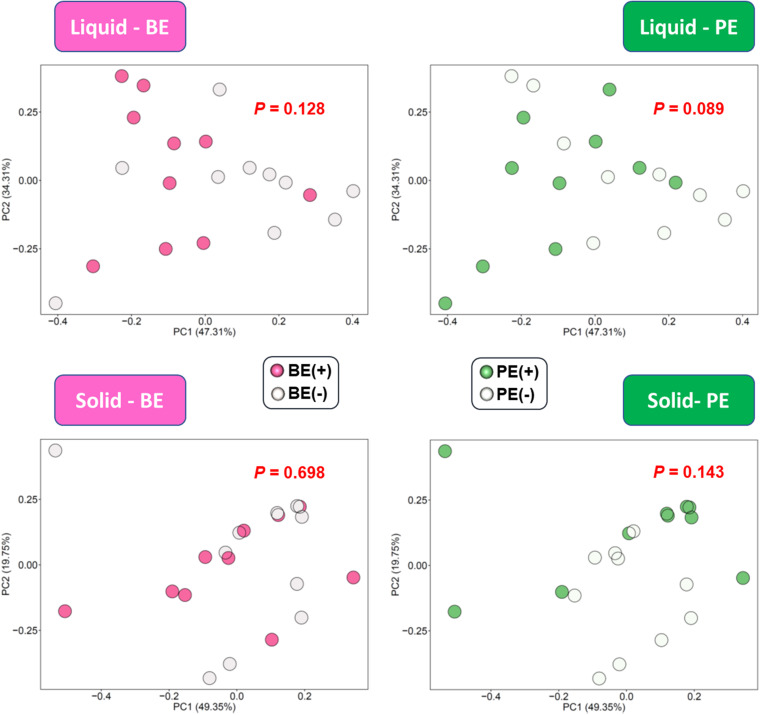
PCA plot showing the effect of microbial inoculations on the overall functional distribution at rumen liquid and solid fractions. Overall functional distribution was computed based on the relative abundance of KEGG orthologs predicted by PICRUSt2.

The overall distribution of microbial functions represented by KEGG ortholog profiles tended to be affected by PE in rumen liquid (*P* = 0.089; Figure 4). Two ruminal fractions had significantly different overall distribution of microbial functions from one another (*P* < 0.001; Supplementary Figure 3). Among the major KEGG pathways, which have over 0.1% average relative abundance either in treated or non-treated calves, eight pathways [“Glycolysis/Gluconeogenesis (ko00010),” “Citrate cycle (TCA cycle) (ko00240),” “Valine, leucine and isoleucine degradation (ko00280),” “Lysine degradation (k00310)”, “D-Alanine metabolism (ko00473),” “Amino sugar and nucleotide sugar metabolism (ko00520),” “Aminoacyl-tRNA biosynthesis (ko00970),” and “Nucleotide excision repair (ko03420)”] were enriched in rumen liquid of BE(+) calves and five pathways [“Ascorbate and aldarate metabolism (ko00053),” “Tyrosine metabolism (ko00350),” “Phenylalanine metabolism (ko00360,” “C5-Branched dibasic acid metabolism (ko00660),” and “Bacterial secretion system (ko03070)”] were enriched in rumen liquid of BE(−) calves (Table 5). In the rumen solid fraction, a total of three pathways [“Selenocompound metabolism (ko00450),” “Glycerophospholipid metabolism (ko00564),” and “Aminoacyl-tRNA biosynthesis (ko00970)” in BE(+) calves] were differentially abundant by BE. In both the rumen fractions, PE(+) calves were more enriched with the “Fatty acid degradation (ko00071)” pathway, while PE(−) calves had a greater abundance of “Pantothenate and CoA biosynthesis (ko00770)”, “Sulfur metabolism (ko00920)”, and “Biofilm formation - Vibrio cholerae (ko05111)” pathways (Table 6). The solid fraction of PE(+) calves exclusively had seven more metabolism-related pathways [“Lysine degradation (ko00310),” “N-Glycan biosynthesis (ko00510),” “Other glycan degradation (ko00511),” “Streptomycin biosynthesis (ko00521),” “Inositol phosphate metabolism (ko00562),” “Methane metabolism (ko00680),” and “Biosynthesis of vancomycin group antibiotics (ko01055)”], whereas two pathways in rumen liquid [“Vitamin B6 metabolism (ko00750)” and “RNA degradation (ko03018)”] and four additional pathways in rumen solids [“Fatty acid biosynthesis (ko00061),” “Secondary bile acid biosynthesis (ko00121),” “Lysine biosynthesis (ko00300),” and “Phosphotransferase system (PTS) (ko02060)”] were differentially abundant in PE(−) treated calves. A total of 19 and 32 major pathways were enriched in rumen liquid and solid, respectively (Supplementary Table 6).

**TABLE 5:** Differentially abundant KEGG pathways by microbial inoculations in liquid fraction.

BE(+) enriched						
KEGG pathways	Relative abundance (%)		SEM	*P*-value	Description	KEGG classification
	BE(+)	BE(−)				
ko00010	1.168	1.101	0.0143	0.019	Glycolysis/Gluconeogenesis	Carbohydrate metabolism
ko00240	1.345	1.292	0.0138	0.041	Pyrimidine metabolism	Nucleotide metabolism
ko00280	0.243	0.230	0.0054	0.041	Valine, leucine and isoleucine degradation	Amino acid metabolism
ko00310	0.103	0.084	0.0055	0.049	Lysine degradation	Amino acid metabolism
ko00473	1.726	1.681	0.0116	0.023	D-Alanine metabolism	Metabolism of other amino acids
ko00520	1.219	1.147	0.0132	0.004	Amino sugar and nucleotide sugar metabolism	Carbohydrate metabolism
ko00970	1.837	1.788	0.0133	0.034	Aminoacyl-tRNA biosynthesis	Translation
ko03420	0.897	0.853	0.0107	0.041	Nucleotide excision repair	Replication and repair

**BE(−) enriched**						
**KEGG pathways**	**Relative abundance (%)**		**SEM**	***P*-value**	**Description**	**KEGG classification**
	**BE(+)**	**BE(−)**				

ko00053	0.233	0.258	0.0061	0.049	Ascorbate and aldarate metabolism	Carbohydrate metabolism
ko00350	0.267	0.280	0.0034	0.034	Tyrosine metabolism	Amino acid metabolism
ko00360	0.309	0.330	0.0048	0.016	Phenylalanine metabolism	Amino acid metabolism
ko00660	1.865	2.067	0.0518	0.049	C5-Branched dibasic acid metabolism	Carbohydrate metabolism
ko03070	0.787	0.831	0.0111	0.023	Bacterial secretion system	Membrane transport

**PE(+) enriched**						
**KEGG pathways**	**Relative abundance (%)**		**SEM**	***P*-value**	**Description**	**KEGG classification**
	**PE(+)**	**PE(−)**				

ko00071	0.294	0.132	0.0291	0.008	Fatty acid degradation	Lipid metabolism

**PE(−) enriched**						
**KEGG pathways**	**Relative abundance (%)**		**SEM**	***P*-value**	**Description**	**KEGG classification**
	**PE(+)**	**PE(−)**				

ko00750	1.509	1.673	0.0409	0.049	Vitamin B6 metabolism	Metabolism of cofactors and vitamins
ko00770	1.769	1.848	0.0166	0.016	Pantothenate and CoA biosynthesis	Metabolism of cofactors and vitamins
ko00920	0.745	0.797	0.0098	0.002	Sulfur metabolism	Energy metabolism
ko03018	0.648	0.693	0.0108	0.041	RNA degradation	Folding, sorting and degradation
ko05111	0.287	0.394	0.0250	0.034	Biofilm formation - Vibrio cholerae	Cellular community - prokaryotes

*KEGG pathways with less than 0.1% average relative abundance in both the treatments were filtered out.*

*BE, bacterial-enriched rumen fluid.*

*PE, protozoal-enriched rumen fluid.*

*SEM, standard error or the mean.*

**TABLE 6:** Differentially abundant KEGG pathways by microbial inoculations in solid fraction.

BE(+) enriched						
**KEGG pathways**	**Relative abundance (%)**		**SEM**	***P*-value**	**Description**	**KEGG classification**
	**BE(+)**	**BE(−)**				

ko00450	0.992	0.957	0.0082	0.028	Selenocompound metabolism	Metabolism of other amino acids
ko00564	0.577	0.561	0.0037	0.028	Glycerophospholipid metabolism	Lipid metabolism
ko00970	1.877	1.830	0.0164	0.034	Aminoacyl-tRNA biosynthesis	Translation

**PE(+) enriched**						
**KEGG pathways**	**Relative abundance (%)**		**SEM**	***P*-value**	**Description**	**KEGG classification**
	**PE(+)**	**PE(−)**				

ko00071	0.307	0.216	0.0305	0.017	Fatty acid degradation	Lipid metabolism
ko00310	0.124	0.108	0.0035	0.010	Lysine degradation	Amino acid metabolism
ko00510	0.101	0.086	0.0029	0.013	N-Glycan biosynthesis	Glycan biosynthesis and metabolism
ko00511	1.825	1.636	0.0616	0.023	Other glycan degradation	Glycan biosynthesis and metabolism
ko00521	1.985	1.868	0.0218	0.005	Streptomycin biosynthesis	Biosynthesis of other secondary metabolites
ko00562	0.267	0.128	0.0320	0.013	Inositol phosphate metabolism	Carbohydrate metabolism
ko00680	0.448	0.416	0.0099	0.041	Methane metabolism	Energy metabolism
ko01055	2.547	2.459	0.0199	0.041	Biosynthesis of vancomycin group antibiotics	Metabolism of terpenoids and polyketides

**PE(−) enriched**						
**KEGG pathways**	**Relative abundance (%)**		**SEM**	***P*-value**	**Description**	**KEGG classification**
	**PE(+)**	**PE(−)**				

ko00061	1.394	1.528	0.0274	0.019	Fatty acid biosynthesis	Lipid metabolism
ko00121	0.181	0.238	0.0244	0.049	Secondary bile acid biosynthesis	Lipid metabolism
ko00300	1.622	1.670	0.0107	0.019	Lysine biosynthesis	Amino acid metabolism
ko00770	1.763	1.828	0.0152	0.013	Pantothenate and CoA biosynthesis	Metabolism of cofactors and vitamins
ko00920	0.766	0.796	0.0097	0.049	Sulfur metabolism	Energy metabolism
ko02060	0.213	0.313	0.0202	0.010	Phosphotransferase system (PTS)	Membrane transport
ko05111	0.244	0.302	0.0141	0.013	Biofilm formation - Vibrio cholerae	Cellular community - prokaryotes

*KEGG pathways with less than 0.1% average relative abundance in both the treatments were filtered out.*

*BE, bacterial-enriched rumen fluid.*

*PE, protozoal-enriched.*

*SEM, standard error or the mean.*

### Correlations Between Bacterial Genera, Animal, and Fermentation Measurements

**FIGURE 5:**
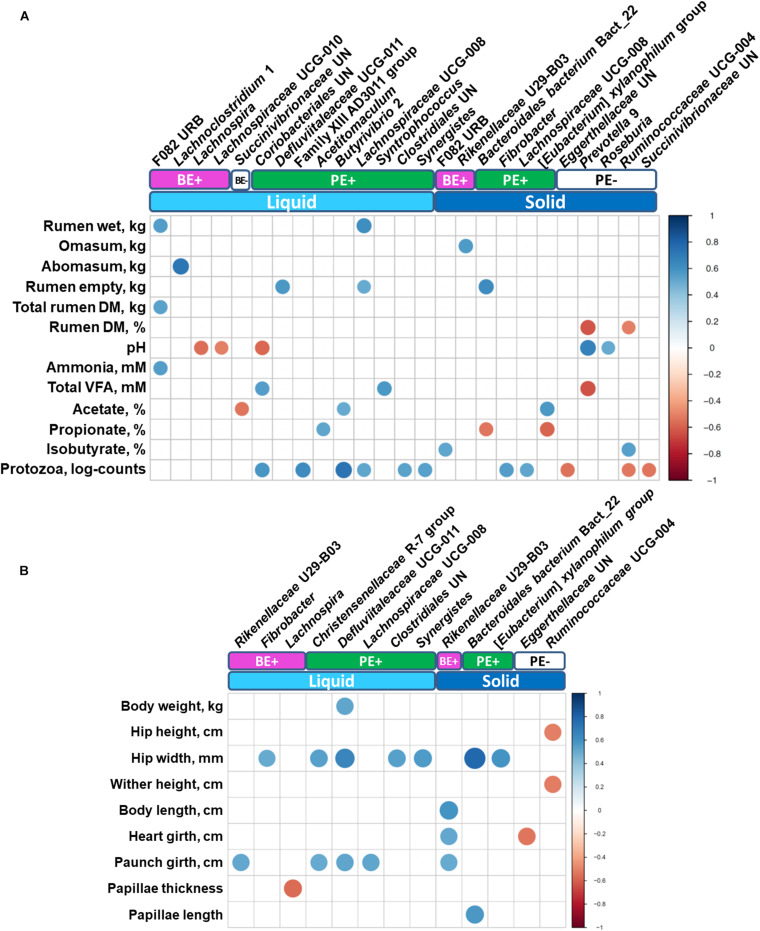
Correlations between **(A)** rumen measurements (stomach compartments, fermentation, and protozoal counts) and **(B)** animal performance measurements with differentially abundant bacterial genera. Only strong significant correlation coefficients (| *r*| ≥ 0.5, *P* ≤ 0.05) were shown on the plot.

Correlation analysis identified various differentially abundant bacterial genera correlated (correlation coefficient, |*r*| ≥ 0.5; *P* ≤ 0.05) with either rumen (Figure 5A) or animal performance measurements (Figure 5B). Unclassified genus of Coriobacteriales and *Syntrophococcus*, which were abundant in PE(+) calves in the liquid fraction, were positively correlated with total VFA concentration, while a negative correlation was found with *Prevotella* 9 enriched in PE(−) calves in the solid fraction. No bacterial taxa showed significant correlations with butyrate molar proportions, however, F082 URB and Ruminococcaceae UCG-004 showed significant positive correlation with isobutyrate molar proportions whose dominance was found in rumen solid fractions of calves treated with autoclaved rumen fluid. Particularly with log_10_-transformed counts of rumen protozoa, eight genera enriched by PE showed significant positive correlations. Three genera dominant in PE(−) calves in the solid fraction (Unclassified genus of Eggerthellaceae, Ruminococcaceae UCG-004, and unclassified genus of Succinivibrionaceae) were negatively correlated with protozoal counts. Bacteroidales bacterium Bact_22 was positively correlated with rumen papillae length, while the bacterial genus *Lachnospira* was negatively correlated with papillae thickness.

## Discussion

Rumen fluid inoculation was applied to direct the early establishment of the rumen microbial community of dairy calves. Dairy calves have much simpler rumen microbiota before weaning than that of post-weaning, but its composition has shown rapid changes by the onset of solid feeding (Jami et al., 2013; Rey et al., 2014). Other researchers have administered microbial inocula during a similar feeding period to enable young ruminants to establish a more diverse microbial community (Belanche et al., 2020; Bu et al., 2020; Yu et al., 2020), which is suggested to facilitate the utilization of complex lignocellulosic contents in the rumen (Ribeiro et al., 2017). In our previous study with pre-weaned dairy calves (3–6 weeks of age), we confirmed that repeated rumen fluid inoculation moderately affected ruminal fermentation parameters, bacterial diversity measurements and composition (Cersosimo et al., 2019). However, sustainability of the inoculation effects on the ruminal microbial community post weaning has not been well demonstrated. This study analyzed rumen bacterial community structures and the performance of post-weaned dairy calves, which were treated with BE and PE inocula before weaning to determine if the previous inoculation effects are detectable.

The increased alpha-diversity measurements observed in post-weaned calves treated with the combined PE/BE inoculum in the present study, particularly in rumen liquid fraction, was not observed in our previous study (Cersosimo et al., 2019). This is possibly due to the growth of transferred microbial taxa in the post-weaned dairy calves fed increased amounts of solids. The immediate establishment of transferred microbiota was not likely (Bryant and Small, 1960) and the inoculation effect conferred by newly transferred microbial taxa could be delayed. The BE increased species richness and phylogenetic diversity in both the solid and liquid fractions, whereas the effect of PE on alpha-diversity measurements was more pronounced in the liquid fraction of treated calves. This might be due to the colonization of the first protozoal group, small entodinia, which are known to prey on bacteria and prefer starch in the rumen (Williams and Coleman, 1992; Newbold et al., 2015). However, the increased alpha-diversity measurements did not result in the significant differences on overall bacterial community structure when the abundance of bacterial taxa were combined. In addition to the different bacterial community structure by quantitative assessment between PE(+) and PE(−) calves in the solid fraction (*P* = 0.063), more distinguishable changes of beta-diversity were observed by qualitative assessment in both of the fractions by PE (*P* < 0.05). This might be due to the transfer of protozoa-associated microbiota which contributed to greater species richness and phylogenetic diversity in PE-inoculum than those of BE-inoculum.

Although not considered as major taxa in this study, the phyla and genera exclusively detected in both of the inoculum and inoculum-treated calves were possibly established by inoculations. Kiritimatiellaeota was recently diverged from the phylum Verrucomicrobia (Spring et al., 2016). Microbiota from this phylum were previously detected in anaerobic conditions, like the animal gut, and could potentially utilize xylose, but no other types of sugars (Spring et al., 2016). The presence of this phylum solely in the solid fraction could indicate an increase in hemicellulose digestion in the rumen of dairy calves. Patescibacteria was observed in a variety of anoxic environments (Herrmann et al., 2019), but because this metagenomically identified phylum has not been well characterized in the rumen, their role in the early rumen is still unknown. In our previous study with the same calves at earlier ages (from 3–6 weeks of age), the abundance of Erysipelotrichaceae unclassified genus linearly increased by age (Cersosimo et al., 2019). Some species belonging to the family Erysipelotrichaceae are saccharolytic and produce lactate by fermenting a variety of sugars (Deusch et al., 2017), which in turn could lower the ruminal pH of inoculum treated calves, especially in BE(+) (*P* < 0.1). While Family XIII AD3011 group was exclusively found in rumen liquid of PE(+) calves as a major bacterial genus, not much information is known for this candidate genus. The establishment of those two tentative genera was previously demonstrated by repeated rumen fluid inoculation of lambs (Yu et al., 2020).

The increased diversity and the qualitatively altered rumen liquid bacterial community structure by PE tended to affect overall functional differences based on the KEGG orthologs profile.

Fatty acid degradation (ko00071) consists of two modules related to beta-oxidation. This pathway was enriched by PE(+) in both of the rumen fractions, so that increasing the yield of acetyl-CoA might be further used to produce energy for cellular biosynthesis in PE treated calves (Goepfert and Poirier, 2007).

Monophyletic Fibrobacteres and Proteobacteria showed opposite distributions by microbial inoculations in the liquid fraction by BE and in the solid fraction by PE. Increased abundance of the genus *Fibrobacter* was observed after faunation of defaunated Holstein bull calves (Ozutsumi et al., 2006). This solid-enriched bacteria, represented over 3% of relative abundance in the solid fraction of PE(+) calves and was suspected to contribute to an increase in fiber digestion resulting in tendency for higher VFA concentration in PE(+) than PE (−) calves (*P* = 0.052). The abundance of the phylum Proteobacteria decreased with age in dairy calves especially after the onset of solid feeding (Jami et al., 2013). Inoculation of dairy calves with adult-derived rumen fluid enriched with Bacteroidetes and Firmicutes in pre-weaned dairy calves might facilitate this transition by lowering the abundance of Proteobacteria.

The unclassified genus of the bacterial family Succinivibrionaceae was a representative genus within Proteobacteria whose abundance was more enriched in the liquid fraction of BE(−) and in the solid fraction of PE(−) calves compared to their respective controls. A 4-fold decrease in the relative abundance of the genus *Succinivibrio* was previously observed between 2 month and 6 month old dairy calves (Jami et al., 2013). The unclassified genus of Rikenellaceae, which was differentially abundant by BE in both of the fractions, is generally considered as primary saccharolytic in many ecosystems and utilizes a variety of fermentable sugars which could have resulted in slightly lowered ruminal pH in BE(+) calves. The known butyrate producers including *Butyrivibrio* 2, *Coprococcus* 1, and Lachnospiraceae UCG-008 were differentially abundant in PE(+) calves in the liquid fraction while only *Roseburia*, as a butyrate-producing bacterium (Vos et al., 2011), was abundant in PE(−) calves in the solid fraction. The lower butyrate proportions in PE(+) calves might be explained by two possible scenarios: 1. a greater abundance of acetogenic bacteria (*Acetitomaculum* and *Syntrophococcus*), or 2. a greater abundance of fibrolytic bacteria, which produce more VFA but not butyrate, inversely lowered the proportion of butyrate in PE(+) calves. Collectively, microbial inoculations facilitated the transition from the Proteobacteria-enriched microbiota to the microbiota occupied by a greater number of bacterial species which can enhance fiber digestion in the rumen. However, opposite distribution of known butyrate producers in between PE(+) and PE(−) calves (*e*.*g*., *Butyrivibrio* 2, *Coprococcus* 1, and Lachnospiraceae UCG-008) calls into question these explanations of the significantly different butyrate molar proportions by PE. Analyzing active microbiota using transcriptomic approaches could help fill the gaps between rumen fermentation and these microbial compositions.

Rumen microbiota produce B vitamins, including pantothenic acid for the host or other microorganisms (Schlau et al., 2012). Several vitamin metabolism enriched pathways were previously reported to be related to high residual feed intake in beef cattle (Li and Guan, 2017) and might be associated with the enrichment of the lactate-utilizers, which require vitamins for their growth (Ogunade et al., 2019). The abundance of unclassified genus of Succinivibrionaceae in the solid fraction of PE(−) calves may also be associated with increased vitamin metabolism. A relatively greater abundance of the biofilm formation pathway in both rumen fractions of PE(−) calves might be related to the absence of rumen protozoa which affect overall composition and activities of rumen microflora by their bacterial predation and close metabolic association particularly with symbionts (Belanche et al., 2014; Park and Yu, 2018).

Exclusive keystone microbial nodes were defined by two centrality measurements. Among those, the genus *Roseburia* was the only node whose abundance corresponded to both the microbial inoculations and ruminal fractions. *Roseburia* is a known butyrate producer that consumes acetate during the fermentation of carbohydrates (Vos et al., 2011), which might contribute to the difference of butyrate molar proportions by PE inoculation. On the other hand, *Roseburia* only exclusively interacts with four microbial nodes which are not considered as butyrate producers (data not shown).

The single largest classified genus in the rumen, *Prevotella*, was also defined as an important exclusive microbial node in the liquid fraction of BE(+) calves. This genus consists of several uncultured *Prevotella* spp. which need further investigation of their metabolism. *Prevotella* usually are considered as multi-functional, utilizing a variety of substrates produced by the rumen microbiome (Bekele et al., 2010). Thus, they might be associated with other microbes whose metabolic functions are diverse. Many exclusive co-occurrence interactions were found with *Eubacterium xylanophilum* group in the solid fraction of BE(+) calves, which might contribute to xylan degradation (Van Gylswyk and Van Der Toorn, 1985). This genus was defined as an important node in the solid fraction of PE(+) calves in this study. Interestingly, the relative abundance of another *Eubacterium* genus, *E. coprostanoligenes* group was increased in lambs inoculated with adult sheep rumen fluid during weaning (Yu et al., 2020).

Within their specific microbial networks altered by microbial inoculations, those bacterial genera might be associated differently with other microbes particularly in different environmental niches. However, since those exclusive nodes only represent 20 to 48.1% of overall bacterial community, the ruminal fermentation and animal phenotypic differences might be derived from the undefined or shared microbial networks occurred in each microbial inoculation treatment.

There were ten and three bacterial genera exhibiting positive and negative correlations with animal performance measurements, respectively. Among those taxa, Bacteroidales bacterium Bact_22 showed positive correlation with papillae length, which could have contributed to the nutrient absorption through the rumen wall (Dehority, 2003; Millen et al., 2016). The order Bacteroidales was previously associated with a greater feed efficiency in steers (Hernandez-Sanabria et al., 2012; Mccann et al., 2014; Myer et al., 2015). Bacterial genera exhibiting significant positive or negative correlations with protozoal log counts might have physical or metabolic associations with rumen protozoa but little is known about any specific relationship. Lachnospiraceae UCG-008, a known butyrate producer, showed positive correlation with the weights of the wet and empty rumen and circumference of paunch girth in addition to the potential association with rumen protozoa in both the ruminal fractions. Future research is required to verify those potential relationships between bacterial taxa and both animal development and ruminal fermentation.

## Conclusion

Compared to our previous results with these calves prior to weaning, a greater number of taxa were shared between treated and non-treated groups. This was also accompanied with increased species richness in groups treated with the microbial inoculations. In PE treated calves, adult-derived rumen inoculations successfully triggered the establishment of some of microbial taxa including entodiniomorphs. In this study, the post-weaned dairy calves, regardless of the microbial inoculations, shared most of the core-bacterial taxa, which predominate in the rumen. Observed differentially abundant taxa and functional pathways by microbial inoculations were associated with a disparity in butyrate proportions by PE, but didn’t result in significant variation in other fermentation measurements or animal development measurements. Lastly, unique microbial networks in each treatment group within different ruminal fractions might contribute to the differential microbial compositions and metabolic shifts as a result of the inoculations.

## Data Availability Statement

The 16S amplicon sequences generated for this study can be found in NCBI Sequence Read Archive, PRJNA454463.

## Ethics Statement

The animal study was reviewed and approved by University of Wisconsin’s Institutional Animal Care and Use Committee. The protocol was approved by the University of Wisconsin’s Institutional Animal Care and Use Committee under the protocol A005829.

## Author Contributions

LC and GZ conceived and designed the study. LC, WR, and GZ conducted the experiment and analyzed the animal data. TP, LC, and WL did the metataxonomic analysis. TP and LC drafted the manuscript. GZ and WL reviewed and edited the manuscript. All the authors read and approved the manuscript.

## Conflict of Interest

The authors declare that the research was conducted in the absence of any commercial or financial relationships that could be construed as a potential conflict of interest.
