# Supplementary figures and images for: Pre-weaning Ruminal Administration of Differentially-Enriched, Rumen-Derived Inocula Shaped Rumen Bacterial Communities and Co-occurrence Networks of Post-weaned Dairy Calves
Source: Front Microbiol. 2021 Feb 26;12:625488. doi: 10.3389/fmicb.2021.625488 (PMC7952535; doi:10.3389/fmicb.2021.625488)

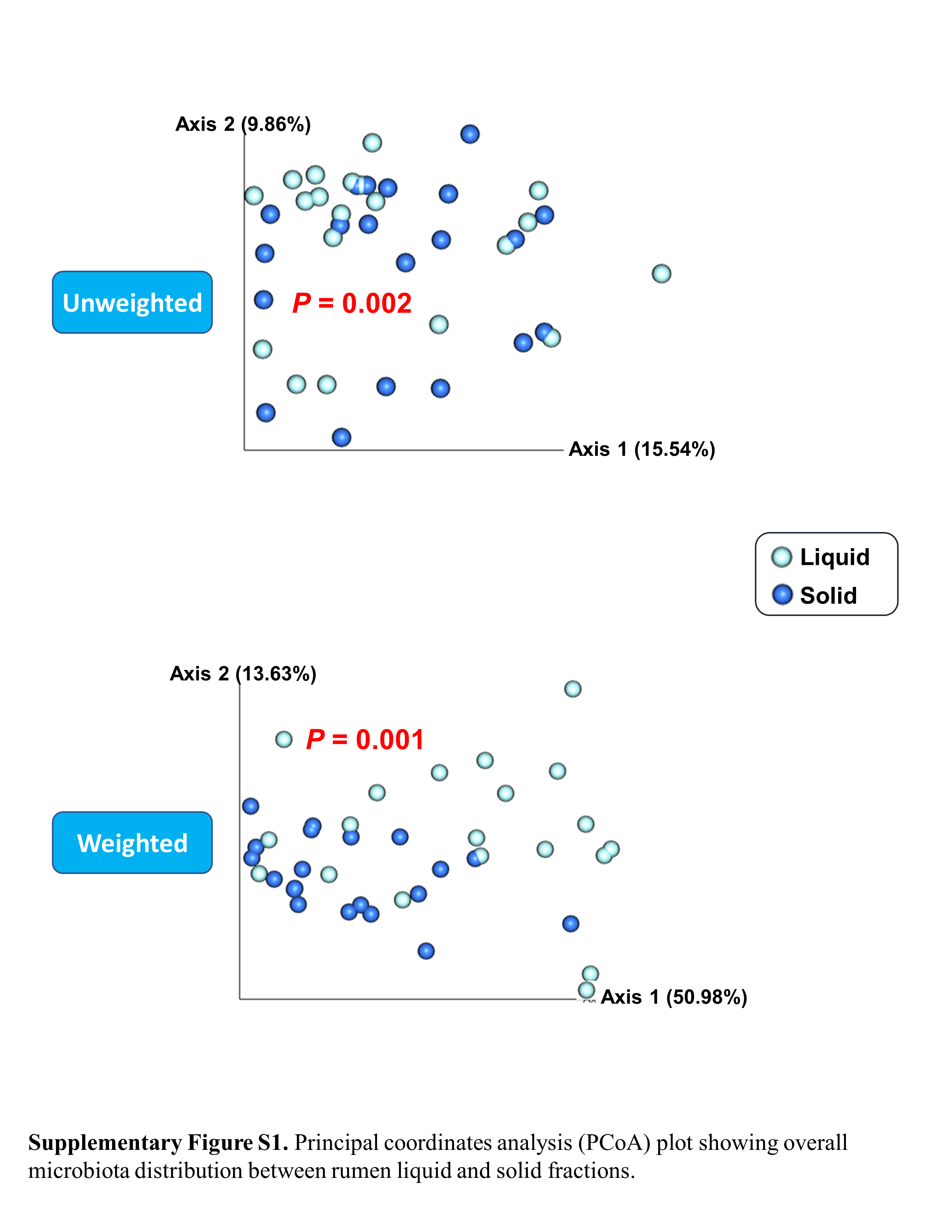

Supplement: Supplementary file 1 [file Image_1.TIF]

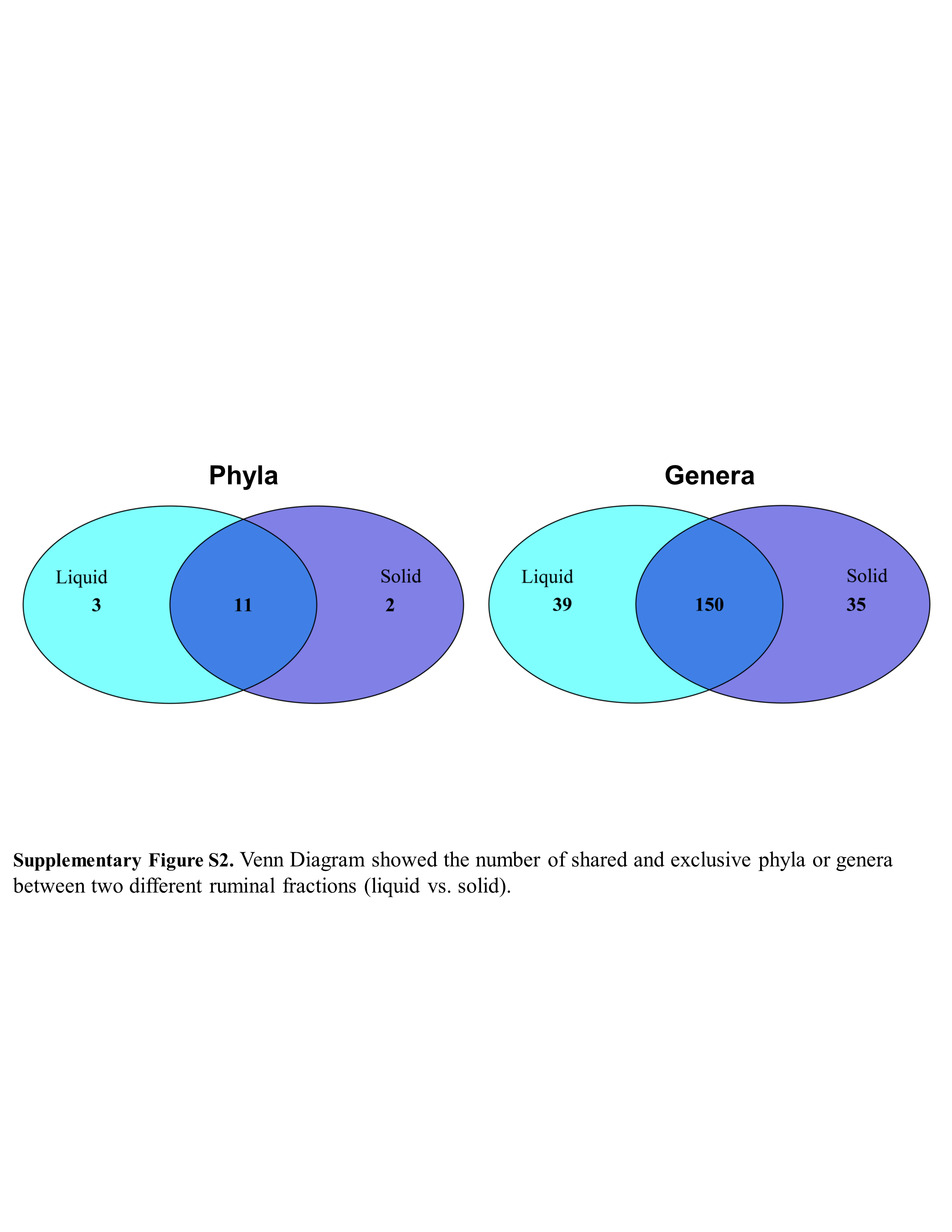

Supplement: Supplementary file 2 [file Image_2.TIF]

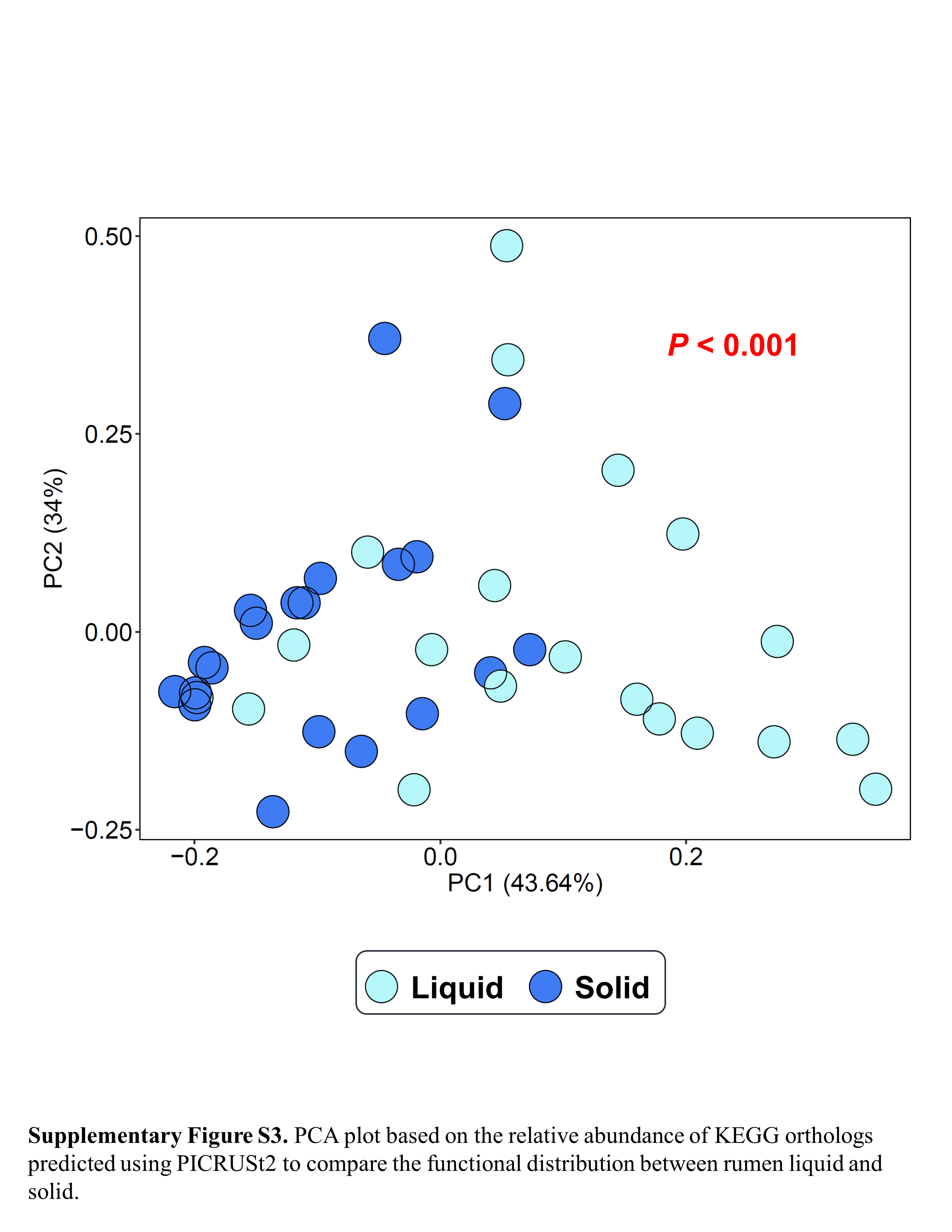

Supplement: Supplementary file 3 [file Image_3.TIF]
