# Supplementary material for: Pre-weaning Ruminal Administration of Differentially-Enriched, Rumen-Derived Inocula Shaped Rumen Bacterial Communities and Co-occurrence Networks of Post-weaned Dairy Calves
Source: Front Microbiol. 2021 Feb 26;12:625488. doi: 10.3389/fmicb.2021.625488 (PMC7952535; doi:10.3389/fmicb.2021.625488)
Supplement: Supplementary file 4 [file Data_Sheet_1.docx]

Supplementary Material

**Pre-weaning ruminal administration of differentially-enriched, rumen-derived inocula shaped rumen bacterial communities and co-occurrence networks of post-weaned dairy calves**

Tansol Park^1,2#*^, Laura M. Cersosimo^1,2#^, Wenli Li^1^, Wendy Radloff^1^, Geoffrey I. Zanton^1*^

^1^USDA-Agricultural Research Service; Dairy Forage Research Center, Madison, WI, USA.

^2^Oak Ridge Institute for Science and Education, Oak Ridge, TN, USA

# These authors equally contributed to this work.

Running head: Sustained effect of adult rumen fluid inoculation in post-weaned dairy calves

* Correspondence note

Geoffrey I. Zanton

Email: [geoffrey.zanton@usda.gov](mailto:geoffrey.zanton@usda.gov)

Tansol Park

Email: [Tansol.Park@usda.gov](mailto:Tansol.Park@usda.gov)

# Supplementary Data

**Supplementary Figure S1.** Principal coordinates analysis (PCoA) plot showing overall microbiota distribution between rumen liquid and solid fractions.

**Supplementary Figure S2.** Venn Diagram showed the number of shared and exclusive phyla or genera between two different ruminal fractions (liquid vs. solid).

**Supplementary Figure S3.** PCA plot based on the relative abundance of KEGG orthologs predicted using PICRUSt2 to compare the functional distribution between rumen liquid and solid.

**Supplementary Table S1.** Animal performance measurements.

| **Measurements** | **BE** | | **PE** | | **SEM** | ***P*-values** | | |
| --- | --- | --- | --- | --- | --- | --- | --- | --- |
|  | **+** | **-** | **+** | **-** |  | **BE** | **PE** | **BE×PE** |
| Body weight (kg) | 103.56 | 98.57 | 103.28 | 98.84 | 2.471 | 0.328 | 0.382 | 0.304 |
| Hip height (cm) | 97.45 | 96.70 | 97.10 | 97.05 | 0.568 | 0.546 | 0.968 | 0.657 |
| Hip width (mm) | 236.8 | 229.9 | 236.3 | 230.4 | 2.659 | 0.214 | 0.285 | 0.956 |
| Wither height (cm) | 92.50 | 91.95 | 92.70 | 91.75 | 0.618 | 0.682 | 0.482 | 0.970 |
| Body length (cm) | 107.4 | 102.3 | 105.6 | 104.1 | 1.545 | 0.119 | 0.634 | 0.924 |
| Heart girth (cm) | 109.05 | 106.05 | 108.30 | 106.80 | 0.971 | 0.129 | 0.436 | 0.302 |
| Paunch girth (cm) | 133.89 | 129.57 | 132.39 | 131.08 | 1.441 | 0.128 | 0.634 | 0.105 |
| Papillae thickness | 1.89 | 2.29 | 2.07 | 2.11 | 0.142 | 0.188 | 0.876 | 0.324 |
| Papillae length | 3.80 | 2.97 | 3.18 | 3.59 | 0.254 | 0.111 | 0.425 | 0.491 |
| Papillae count | 111.8 | 103.8 | 112.6 | 103.0 | 5.328 | 0.477 | 0.396 | 0.757 |
| Lesion score | 6.12 | 6.78 | 6.62 | 6.28 | 0.351 | 0.387 | 0.653 | 0.979 |

BE, bacterial-enriched rumen fluid.

PE, protozoal-enriched rumen fluid.

SEM, standard error of the mean.

**Supplementary Table S2.** Sample information and sequencing statistics of 16S rRNA gene amplicon sequencing variants.

| **Calf ID** | **Fraction** | **No. of input paired reads** | **Quality filtered reads** | **Denoised reads** | **Merged reads** | **Chimera-filtered reads** | **Taxa-filtered*** |
| --- | --- | --- | --- | --- | --- | --- | --- |
| Calf 1 | Liquid | 37,171 | 36,994 | 36,766 | 36,409 | 36,254 | 34,387 |
| Calf 1 | Solid | 21,923 | 21,785 | 21,556 | 21,358 | 21,218 | 20,956 |
| Calf 2 | Liquid | 35,926 | 35,580 | 35,331 | 34,680 | 34,518 | 32,368 |
| Calf 2 | Solid | 21,219 | 21,086 | 20,956 | 20,764 | 20,589 | 20,278 |
| Calf 3 | Liquid | 28,452 | 28,221 | 28,020 | 27,866 | 27,706 | 27,239 |
| Calf 3 | Solid | 22,570 | 22,374 | 22,198 | 22,031 | 21,837 | 21,492 |
| Calf 4 | Liquid | 33,056 | 32,829 | 32,627 | 32,324 | 31,935 | 31,222 |
| Calf 4 | Solid | 26,814 | 26,583 | 26,376 | 26,197 | 25,981 | 25,500 |
| Calf 5 | Liquid | 27,827 | 27,621 | 27,431 | 27,228 | 26,995 | 26,895 |
| Calf 5 | Solid | 30,872 | 30,713 | 30,534 | 30,222 | 29,744 | 29,695 |
| Calf 6 | Liquid | 32,743 | 32,340 | 32,191 | 30,501 | 30,289 | 27,858 |
| Calf 6 | Solid | 20,238 | 20,098 | 19,944 | 19,733 | 19,519 | 19,050 |
| Calf 7 | Liquid | 32,089 | 31,615 | 31,366 | 28,726 | 28,581 | 25,521 |
| Calf 7 | Solid | 17,115 | 16,973 | 16,854 | 16,691 | 16,613 | 15,896 |
| Calf 8 | Liquid | 28,109 | 27,954 | 27,777 | 27,591 | 27,475 | 27,307 |
| Calf 8 | Solid | 19,604 | 19,456 | 19,347 | 19,196 | 18,892 | 18,766 |
| Calf 9 | Liquid | 24,977 | 24,846 | 24,707 | 24,607 | 24,408 | 24,279 |
| Calf 9 | Solid | 54,714 | 54,421 | 54,190 | 53,551 | 51,717 | 51,348 |
| Calf 10 | Liquid | 21,691 | 21,562 | 21,412 | 21,240 | 21,110 | 20,987 |
| Calf 10 | Solid | 24,177 | 24,055 | 23,902 | 23,704 | 23,422 | 22,961 |
| Calf 11 | Liquid | 32,785 | 32,520 | 32,252 | 31,831 | 31,432 | 30,648 |
| Calf 11 | Solid | 23,307 | 23,081 | 22,908 | 22,698 | 22,443 | 21,829 |
| Calf 12 | Liquid | 25,817 | 25,659 | 25,540 | 25,314 | 25,131 | 24,909 |
| Calf 12 | Solid | 26,467 | 26,323 | 26,199 | 25,989 | 25,746 | 25,448 |
| Calf 13 | Liquid | 24,747 | 24,578 | 24,337 | 24,038 | 23,916 | 23,044 |
| Calf 13 | Solid | 23,645 | 23,465 | 23,271 | 23,046 | 22,878 | 21,967 |
| Calf 14 | Liquid | 35,278 | 35,028 | 34,834 | 34,578 | 34,424 | 33,693 |
| Calf 14 | Solid | 19,152 | 18,983 | 18,826 | 18,629 | 18,540 | 18,052 |
| Calf 15 | Liquid | 21,232 | 21,089 | 20,876 | 20,656 | 20,358 | 19,766 |
| Calf 15 | Solid | 34,479 | 34,220 | 34,006 | 33,438 | 33,252 | 32,861 |
| Calf 16 | Liquid | 21,444 | 21,232 | 21,115 | 20,239 | 19,862 | 18,569 |
| Calf 16 | Solid | 20,477 | 20,303 | 20,223 | 20,120 | 20,012 | 19,561 |
| Calf 17 | Liquid | 31,870 | 31,640 | 31,450 | 31,187 | 31,016 | 30,365 |
| Calf 17 | Solid | 22,258 | 22,126 | 22,029 | 21,831 | 21,619 | 21,387 |
| Calf 18 | Liquid | 28,929 | 28,745 | 28,572 | 28,275 | 28,141 | 28,012 |
| Calf 18 | Solid | 16,699 | 16,543 | 16,417 | 16,273 | 16,203 | 16,061 |
| Calf 19 | Liquid | 35,388 | 35,161 | 34,987 | 34,775 | 34,571 | 34,454 |
| Calf 19 | Solid | 22,144 | 21,969 | 21,787 | 21,607 | 21,435 | 21,360 |
| Calf 20 | Liquid | 15,356 | 15,272 | 15,186 | 14,992 | 14,907 | 14,893 |
| Calf 20 | Solid | 21,892 | 21,715 | 21,572 | 21,432 | 21,277 | 21,239 |
| BE | Inoculum | 23,828 | 23,674 | 22,956 | 22,449 | 21,314 | 20,822 |
| PE | Inoculum | 26,148 | 25,960 | 25,156 | 24,551 | 23,491 | 23,055 |

* Amplicon sequencing variants (ASVs) identified as “Archaea”, “Cyanobacteria”, “mitochondria”, “chloroplast”, and “Unassigned” were filtered out.

BE, bacterial-enriched rumen fluid.

PE, protozoal-enriched rumen fluid.

**Supplementary Table S3.** Alpha diversity between liquid and solid.

| **Diversity measurements** | **Fractions** | | **SEM** | ***P*-value** |
| --- | --- | --- | --- | --- |
|  | **Liquid** | **Solid** |  |  |
| Observed ASVs | 164 | 156 | 6.0036 | 0.402 |
| Chao1 estimates | 170 | 161 | 6.3718 | 0.329 |
| Evenness | 0.634 | 0.669 | 0.0093 | 0.040 |
| Faith’s phylogenetic diversity | 12.837 | 12.464 | 0.3456 | 0.445 |
| Shannon’s index | 4.650 | 4.858 | 0.0937 | 0.200 |
| Simpson’s index | 0.901 | 0.921 | 0.0068 | 0.116 |

Good’s coverage was >99.8% in all samples.

Calf as random effect.

ASV, amplicon sequencing variant.

SEM, standard error of the mean.

**Supplementary Table S4.** Alpha-diversity measurements in inoculums.

| Inoculum type | Diversity measurements | | | | | |
| --- | --- | --- | --- | --- | --- | --- |
|  | Observed ASVs | Chao1 estimates | Evenness | Faith's phylogenetic diversity | Shannon’s index | Simpson’s index |
| Bacteria-enriched inoculum | 535 | 564 | 0.831 | 32.482 | 7.520 | 0.986 |
| Protozoa-enriched inoculum | 601 | 632 | 0.824 | 36.159 | 7.599 | 0.983 |
| SEM | 16.7330 | 18.0482 | 0.0069 | 0.7750 | 0.0768 | 0.0016 |
| *P*-value | 0.044 | 0.056 | 0.628 | 0.013 | 0.621 | 0.405 |

Minimum Good’s coverage > 98.7%.

Those indices were measured from 20 samples (10 sample each) collected during the microbial inoculation at 3 to 6 weeks age of calves.

ASV, amplicon sequencing variant.

SEM, standard error of the mean.

**Supplementary Table S5.** Differentially abundant taxa (phyla and genera) between rumen liquid and solid fraction.

| **Enrichment** | **Phyla** | **Relative abundance (%)** | | **SEM** | ***P*-value** |
| --- | --- | --- | --- | --- | --- |
|  |  | **Liquid** | **Solid** |  |  |
| Liquid-enriched | *Proteobacteria* | 21.092 | 6.615 | 2.1807 | <0.001 |
| Solid-enriched | *Bacteroidetes* | 46.633 | 58.943 | 1.8957 | 0.002 |
|  | *Fibrobacteres* | 0.133 | 2.012 | 0.3204 | 0.001 |
|  | *Spirochaetes* | 0.050 | 1.406 | 0.2909 | 0.001 |

| **Enrichment** | **Phyla** | **Genera** | **Relative abundance (%)** | | **SEM** | ***P*-value** |
| --- | --- | --- | --- | --- | --- | --- |
|  |  |  | **Liquid** | **Solid** |  |  |
| Liquid-enriched | *Bacteroidetes* | *Prevotellaceae* UN | 1.536 | 1.126 | 0.1436 | 0.045 |
|  |  | *Bacteroidales* UN | 0.154 | 0.083 | 0.0171 | 0.020 |
|  | *Firmicutes* | *Christensenellaceae* R-7 group | 0.087 | 0.045 | 0.0131 | 0.007 |
|  |  | *Lachnospira* | 0.069 | 0.031 | 0.0071 | 0.007 |
|  |  | *Ruminococcaceae* NK4A214 group | 0.213 | 0.044 | 0.0281 | <0.001 |
|  |  | *Eubacterium* *coprostanoligenes* group | 0.478 | 0.274 | 0.0380 | 0.009 |
|  |  | *Ruminococcaceae* UN | 0.026 | 0.014 | 0.0038 | 0.017 |
|  |  | *Erysipelotrichaceae* UCG-002 | 1.814 | 0.484 | 0.2326 | 0.028 |
|  |  | *Acidaminococcus* | 1.267 | 0.868 | 0.0970 | 0.025 |
|  |  | *Megasphaera* | 0.628 | 0.107 | 0.0870 | 0.004 |
|  |  | *Veillonellaceae* uncultured | 1.238 | 0.039 | 0.3023 | <0.001 |
|  |  | *Veillonellaceae* UN | 1.032 | 0.317 | 0.1383 | 0.002 |
|  | *Proteobacteria* | *Desulfovibrio* | 0.157 | 0.034 | 0.0167 | <0.001 |
|  |  | *Succinivibrionaceae* UCG-001 | 9.652 | 0.785 | 1.1340 | <0.001 |
| Solid-enriched | *Bacteroidetes* | F082 URB | 0.124 | 0.519 | 0.0661 | 0.015 |
|  |  | *Prevotella* 7 | 37.391 | 47.839 | 2.0532 | 0.012 |
|  |  | *Prevotella* sp. AN 5135 | 0.999 | 1.794 | 0.2139 | 0.028 |
|  |  | *Prevotellaceae* UCG-001 | 0.571 | 1.406 | 0.1885 | 0.030 |
|  |  | *Prevotellaceae* UCG-004 | 0.049 | 0.227 | 0.0383 | 0.002 |
|  |  | *Rikenellaceae* RC9 gut group | 0.961 | 2.587 | 0.3601 | 0.008 |
|  | *Fibrobacteres* | *Fibrobacter* | 0.133 | 2.012 | 0.3204 | 0.001 |
|  | *Firmicutes* | *Mogibacterium* | 0.016 | 0.043 | 0.0053 | 0.050 |
|  |  | *Oribacterium* | 1.103 | 2.798 | 0.3458 | 0.006 |
|  |  | *Eubacterium* *ruminantium* group | 0.016 | 0.560 | 0.1525 | 0.014 |
|  |  | *Eubacterium* *xylanophilum* group | 0.012 | 0.075 | 0.0160 | 0.028 |
|  |  | *Erysipelotrichaceae* UCG-004 | 0.034 | 0.155 | 0.0208 | <0.001 |
|  |  | *Dialister* | 3.191 | 4.874 | 0.3700 | 0.019 |
|  | *Spirochaetes* | *Treponema* 2 | 0.046 | 1.391 | 0.2897 | 0.001 |

* Calculated based on the normalized read count BIOM tables.

UN, unclassified; URB, uncultured rumen bacterium, UCG, uncultured genus-level group.

SEM, standard error or the mean.

**Supplementary Table S6.** Enriched KEGG pathways between rumen liquid and solid fraction.

| **Liquid-enriched** | | | | | | |
| --- | --- | --- | --- | --- | --- | --- |
| KEGG pathways | Relative abundance (%) | | SEM | *P*-value | Description | KEGG classification |
|  | Liquid | Solid |  |  |  |  |
| ko00053 | 0.246 | 0.200 | 0.0052 | <0.001 | Ascorbate and aldarate metabolism | Carbohydrate metabolism |
| ko00061 | 1.609 | 1.461 | 0.0283 | 0.021 | Fatty acid biosynthesis | Lipid metabolism |
| ko00350 | 0.274 | 0.257 | 0.0024 | <0.001 | Tyrosine metabolism | Amino acid metabolism |
| ko00440 | 0.139 | 0.100 | 0.0059 | 0.001 | Phosphonate and phosphinate metabolism | Metabolism of other amino acids |
| ko00480 | 0.660 | 0.569 | 0.0149 | 0.003 | Glutathione metabolism | Metabolism of other amino acids |
| ko00561 | 0.454 | 0.410 | 0.0078 | 0.003 | Glycerolipid metabolism | Lipid metabolism |
| ko00564 | 0.592 | 0.569 | 0.0040 | 0.001 | Glycerophospholipid metabolism | Lipid metabolism |
| ko00620 | 1.029 | 0.959 | 0.0104 | 0.001 | Pyruvate metabolism | Carbohydrate metabolism |
| ko00630 | 0.591 | 0.547 | 0.0063 | 0.001 | Glyoxylate and dicarboxylate metabolism | Carbohydrate metabolism |
| ko00640 | 0.546 | 0.478 | 0.0243 | 0.004 | Propanoate metabolism | Carbohydrate metabolism |
| ko00650 | 0.628 | 0.591 | 0.0051 | <0.001 | Butanoate metabolism | Carbohydrate metabolism |
| ko00780 | 1.188 | 0.984 | 0.0391 | 0.015 | Biotin metabolism | Metabolism of cofactors and vitamins |
| ko02010 | 0.526 | 0.468 | 0.0136 | 0.009 | ABC transporters | Membrane transport |
| ko02020 | 0.286 | 0.254 | 0.0078 | 0.048 | Two-component system | Signal transduction |
| ko02040 | 1.131 | 0.811 | 0.0542 | 0.007 | Flagellar assembly | Cell motility |
| ko02060 | 0.432 | 0.263 | 0.0207 | <0.001 | Phosphotransferase system (PTS) | Membrane transport |
| ko03070 | 0.809 | 0.770 | 0.0071 | 0.007 | Bacterial secretion system | Membrane transport |
| ko04122 | 0.868 | 0.615 | 0.0312 | <0.001 | Sulfur relay system | Folding, sorting and degradation |
| ko05111 | 0.341 | 0.273 | 0.0152 | 0.042 | Biofilm formation - Vibrio cholerae | Cellular community - prokaryotes |
| **Solid-enriched** | | | | | | |
| KEGG pathways | Relative abundance (%) | | SEM | *P*-value | Description | KEGG classification |
|  | Liquid | Solid |  |  |  |  |
| ko00040 | 0.578 | 0.609 | 0.0169 | 0.040 | Pentose and glucuronate interconversions | Carbohydrate metabolism |
| ko00071 | 0.213 | 0.262 | 0.0212 | 0.015 | Fatty acid degradation | Lipid metabolism |
| ko00190 | 0.494 | 0.535 | 0.0066 | 0.002 | Oxidative phosphorylation | Energy metabolism |
| ko00240 | 1.318 | 1.363 | 0.0098 | 0.023 | Pyrimidine metabolism | Nucleotide metabolism |
| ko00250 | 1.874 | 1.941 | 0.0110 | 0.002 | Alanine, aspartate and glutamate metabolism | Amino acid metabolism |
| ko00260 | 1.215 | 1.245 | 0.0065 | 0.015 | Glycine, serine and threonine metabolism | Amino acid metabolism |
| ko00310 | 0.094 | 0.116 | 0.0037 | 0.001 | Lysine degradation | Amino acid metabolism |
| ko00330 | 0.692 | 0.735 | 0.0071 | 0.003 | Arginine and proline metabolism | Amino acid metabolism |
| ko00450 | 0.939 | 0.974 | 0.0062 | 0.006 | Selenocompound metabolism | Metabolism of other amino acids |
| ko00471 | 2.487 | 2.576 | 0.0211 | 0.001 | D-Glutamine and D-glutamate metabolism | Metabolism of other amino acids |
| ko00473 | 1.704 | 1.767 | 0.0106 | <0.001 | D-Alanine metabolism | Metabolism of other amino acids |
| ko00500 | 0.993 | 1.103 | 0.0173 | 0.001 | Starch and sucrose metabolism | Carbohydrate metabolism |
| ko00511 | 1.351 | 1.730 | 0.0611 | 0.002 | Other glycan degradation | Glycan biosynthesis and metabolism |
| ko00521 | 1.743 | 1.927 | 0.0232 | <0.001 | Streptomycin biosynthesis | Biosynthesis of other secondary metabolites |
| ko00531 | 0.421 | 0.538 | 0.0210 | 0.003 | Glycosaminoglycan degradation | Glycan biosynthesis and metabolism |
| ko00550 | 2.188 | 2.241 | 0.0167 | 0.021 | Peptidoglycan biosynthesis | Glycan biosynthesis and metabolism |
| ko00670 | 2.128 | 2.275 | 0.0267 | 0.005 | One carbon pool by folate | Metabolism of cofactors and vitamins |
| ko00710 | 1.953 | 2.033 | 0.0152 | 0.006 | Carbon fixation in photosynthetic organisms | Energy metabolism |
| ko00720 | 1.221 | 1.254 | 0.0103 | 0.048 | Carbon fixation pathways in prokaryotes | Energy metabolism |
| ko00900 | 1.544 | 1.632 | 0.0134 | <0.001 | Terpenoid backbone biosynthesis | Metabolism of terpenoids and polyketides |
| ko00908 | 0.913 | 1.045 | 0.0192 | 0.001 | Zeatin biosynthesis | Metabolism of terpenoids and polyketides |
| ko00910 | 0.623 | 0.661 | 0.0060 | 0.002 | Nitrogen metabolism | Energy metabolism |
| ko00970 | 1.812 | 1.854 | 0.0109 | 0.001 | Aminoacyl-tRNA biosynthesis | Translation |
| ko01055 | 2.424 | 2.503 | 0.0168 | 0.017 | Biosynthesis of vancomycin group antibiotics | Metabolism of terpenoids and polyketides |
| ko03010 | 1.846 | 1.911 | 0.0156 | 0.001 | Ribosome | Translation |
| ko03020 | 1.156 | 1.287 | 0.0171 | <0.001 | RNA polymerase | Transcription |
| ko03420 | 0.875 | 0.940 | 0.0087 | <0.001 | Nucleotide excision repair | Replication and repair |
| ko03430 | 1.887 | 1.939 | 0.0126 | 0.017 | Mismatch repair | Replication and repair |
| ko04112 | 1.712 | 1.785 | 0.0115 | <0.001 | Cell cycle - Caulobacter | Cell growth and death |
| ko04210 | 0.080 | 0.101 | 0.0042 | 0.005 | Apoptosis | Cell growth and death |
| ko04974 | 0.094 | 0.126 | 0.0048 | 0.001 | Protein digestion and absorption | Digestive system |
| ko05120 | 0.180 | 0.184 | 0.0008 | 0.033 | Epithelial cell signaling in Helicobacter pylori infection | Infectious diseases: Bacterial |

SEM, standard error or the mean.
